# Association of the Mandatory Medicare Bundled Payment With Joint Replacement Outcomes in Hospitals With Disadvantaged Patients

**DOI:** 10.1001/jamanetworkopen.2019.14696

**Published:** 2019-11-06

**Authors:** Hyunjee Kim, Thomas H. A. Meath, Konrad Dobbertin, Ana R. Quiñones, Said A. Ibrahim, K. John McConnell

**Affiliations:** 1 Center for Health Systems Effectiveness, Oregon Health & Science University, Portland; 2 Department of Family Medicine, Oregon Health & Science University, Portland; 3 Department of Healthcare Policy and Research, Weill Cornell Medicine/New York-Presbyterian, New York, New York

## Abstract

**Importance:**

Medicare’s Comprehensive Care for Joint Replacement (CJR) model rewards or penalizes hospitals on the basis of meeting spending benchmarks that do not account for patients’ preexisting social and medical complexity or high expenses associated with serving disadvantaged populations such as dual-eligible patients (ie, those enrolled in both Medicare and Medicaid). The CJR model may have different implications for hospitals serving a high percentage of dual-eligible patients (termed *high-dual*) and hospitals serving a low percentage of dual-eligible patients (termed *low-dual*).

**Objective:**

To examine changes associated with the CJR model among high-dual or low-dual hospitals in 2016 to 2017.

**Design, Setting, and Participants:**

This cohort study comprised 3 analyses of high-dual or low-dual hospitals (n = 1165) serving patients with hip or knee joint replacements (n = 768 224) in 67 treatment metropolitan statistical areas (MSAs) selected for CJR participation and 103 control MSAs. The study used Medicare claims data and public reports from 2012 to 2017. Data analysis was conducted from February 1, 2019, to August 31, 2019.

**Exposures:**

The CJR model holds participating hospitals accountable for the spending and quality of care during care episodes for patients with hip or knee joint replacement, including hospitalization and 90 days after discharge.

**Main Outcomes and Measures:**

The primary outcomes were total episode spending, discharge to institutional postacute care facility, and readmission within the 90-day postdischarge period; bonus and penalty payments for each hospital; and reductions in per-episode spending required to receive a bonus for each hospital.

**Results:**

In total, 1165 hospitals (291 high-dual and 874 low-dual) and 768 224 patients with joint replacement (494 013 women [64.3%]; mean [SD] age, 76 [7] years) were included. An episode-level triple-difference analysis indicated that total spending under the CJR model decreased at high-dual hospitals (by $851; 95% CI, –$1556 to –$146; *P* = .02) and low-dual hospitals (by $567; 95% CI, –$933 to –$202; *P* = .003). The size of decreases did not differ between the 2 groups (difference, –$284; 95% CI, –$981 to $413; *P* = .42). Discharge to institutional postacute care settings and readmission did not change among both hospital groups. High-dual hospitals were less likely to receive a bonus compared with low-dual hospitals (40.3% vs 59.1% in 2016; 56.9% vs 76.0% in 2017). To receive a bonus, high-dual hospitals would be required to reduce spending by $887 to $2231 per episode, compared with only $89 to $215 for low-dual hospitals.

**Conclusions and Relevance:**

The study found that high- and low-dual hospitals made changes in care after CJR implementation, and the magnitude of these changes did not differ between the 2 groups. However, high-dual hospitals were less likely to receive a bonus for spending cuts. Spending benchmarks for CJR would require high-dual hospitals to reduce spending more substantially to receive a financial incentive.

## Key Points

**Question:**

How did care change in hospitals serving a high percentage of disadvantaged patients vs hospitals serving a low percentage under Medicare’s Comprehensive Care for Joint Replacement model?

**Findings:**

In this cohort study of 1165 hospitals serving 768 224 patients who underwent joint replacement, hospitals with a high or low percentage of dual-eligible patients reduced their spending under the Comprehensive Care for Joint Replacement model, but the magnitude of reduction did not differ. Hospitals with a high percentage of disadvantaged patients were required to make more substantial cuts for a bonus and were less likely than other hospitals to receive that bonus.

**Meaning:**

The findings suggest that, under the Comprehensive Care for Joint Replacement system, hospitals with a high percentage of disadvantaged patients must reduce spending more substantially than their counterparts to obtain a financial incentive despite their high share of patients with complex social and medical needs.

## Teaser

This cohort study examines changes in health care spending, use, and quality of care after the implementation of Medicare’s first mandatory bundled payment system at participating hospitals and among dual-eligible patients.

## Introduction

The Medicare program is increasingly focusing on value-based payments to reduce costs and improve quality of care. However, value-based payments may differentially affect hospitals serving a high percentage of patients with low socioeconomic status or medical complexity.^1,2,3^ Patients with social and medical complexity may have needs that are insufficiently measured through traditional risk adjustment, affecting a hospital’s performance on spending and quality measures of value-based payment programs. These concerns may be particularly salient when considering the influence of mandatory value-based payment models on hospitals serving a high percentage of disadvantaged patients.

The Comprehensive Care for Joint Replacement (CJR) model was introduced by Medicare in April 2016 and will continue through 2020. It is Medicare’s first mandatory bundled payment model and was implemented in 67 metropolitan statistical areas (MSAs) selected by the Centers for Medicare & Medicaid Services (CMS) using stratified cluster randomized sampling. All hospitals in the selected MSAs (except those participating in the voluntary Bundled Payments for Care Improvement [BPCI] Initiative) were required to participate. The CJR model holds hospitals accountable for spending and quality of care for patients receiving hip or knee joint replacements during care episodes that include hospitalization and 90-day postdischarge care. If episode spending exceeds the quality-adjusted spending benchmark, hospitals are required to pay a penalty. If spending is below benchmark and hospitals meet the quality threshold, they receive a bonus.^4^

This payment system may have different implications for hospitals serving a high percentage of dual-eligible patients, defined as patients enrolled in both Medicare and Medicaid, who received hip or knee joint replacement.^5^ Episode spending for dual-eligible patients with joint replacement is higher owing to their preexisting social and medical complexity.^5,6,7,8^ However, CJR spending benchmarks do not account for preexisting social and medical complexity. Instead, benchmarks are based on a blend of a hospital’s historical spending and regional historical spending on care episodes and will eventually be completely based on regional historical spending.

This study examined changes in hospitals with a high percentage of dual-eligible patients with joint replacement in comparison with other hospitals with a low percentage under the CJR model in 2016 and 2017. We used percentages of dual-eligible patients to measure hospitals serving a high proportion of disadvantaged patients because this metric had advantages over other measures (eg, hospitals with a high disproportionate patient percentage). For example, the dual-eligible measure better differentiated hospitals serving a high proportion of disadvantaged patients on outcomes common among disadvantaged patients with joint replacement, such as high readmission rates.

This study included 3 analyses of hospitals with a high or low percentage of dual-eligible patients. First, we assessed how spending, health service use, and quality of care changed under the CJR model in these hospitals. Second, we analyzed CJR reconciliation (bonus or penalty) payments for these hospitals. Third, we examined how much reduction in spending would be necessary for these hospitals to earn bonuses under the CJR model. The study aimed to provide insights on care for vulnerable populations under large-scale payment reforms.

## Methods

The institutional review board at the Oregon Health & Science University approved this cohort study with a waiver of informed consent, because seeking informed consent from all patients included in the study was not feasible and the risk to study participants was minimal. Data analysis was conducted from February 1, 2019, to August 31, 2019. This study followed the Strengthening the Reporting of Observational Studies in Epidemiology (STROBE) reporting guideline.

### Study Setting

We selected MSAs for the study through the sampling frame that CMS used for CJR participation (eFigure 1 in the Supplement). First, CMS excluded 192 of 388 MSAs, owing to a low volume of hip or knee joint replacements or high participation rates in the BPCI Initiative. Next, CMS divided the remaining 196 MSAs into 8 strata according to population size and historical spending. In each of these 8 strata, CMS randomly selected MSAs for CJR participation, with higher selection probabilities applied for higher-spending strata. In July 2015, CMS announced 75 treatment MSAs and 121 control MSAs for CJR participation. However, in November 2015, CMS updated the MSA exclusion criteria for the CJR model to account for provider BPCI Initiative participation during July through September 2015. On the basis of the updated exclusion criteria, CMS excluded 8 treatment and 17 control MSAs, leaving 67 treatment and 104 control MSAs. The list of the 67 treatment and 104 control MSAs is available on the CMS website.^4,9^ We excluded 1 additional control MSA in Puerto Rico, which was struck by Hurricane Maria in 2017.

The final sample comprised 67 treatment and 103 control MSAs. Previous studies used an intention-to-treat approach based on the CMS initial sampling frame and included 75 treatment and 121 control groups.^10,11,12^ However, the present study used 67 treatment and 103 control groups based on the CMS final sampling frame and could estimate changes associated with the CJR model more accurately. In the Supplement, eAppendix 1 details the stratified cluster randomized sampling method used by CMS and eFigure 2 displays a map of treatment and control MSAs.

### Data Sources and Sample Selection

We used publicly available data for each hospital’s CJR spending benchmark and reconciliation (bonus or penalty) payments.^4^ We used the Medicare Master Beneficiary Summary File and the inpatient, skilled nursing facility, home health agency, outpatient, and carrier claims from 2012 through 2017 to identify spending, health service use, and quality of care associated with hip or knee joint replacements as well as patient characteristics.^13^ We also used the CMS Provider of Services File for hospital characteristics and Area Health Resources Files for MSA characteristics.^14,15,16^

This study included hip or knee joint replacements (identified by Medicare Severity-Diagnosis Related Group codes 469 and 470 in inpatient claims) that occurred in 67 treatment and 103 control MSAs between 2012 and 2017, except those that occurred during the washout period between January 2015 and March 2016. We excluded joint replacements performed at BPCI Initiative–participating hospitals because these institutions were exempt from CJR participation. We also excluded joint replacements from hospitals that performed fewer than 11 joint replacements each year to obtain reliable percentages of dual-eligible patients in each hospital. We excluded joint replacements for patients who were younger than 66 years or not continuously enrolled in Medicare Part A or B from 1 year before the index admission to 90 days after discharge (except those who died during a care episode). Replacements with any missing information were also excluded. eFigure 3 in the Supplement shows all study sample exclusions.

### Hospitals With a High Percentage of Disadvantaged Patients

We identified hospitals serving a high proportion of disadvantaged patients as those in the top quartile in percentages of dual-eligible patients with hip or knee joint replacement during the pre-CJR period. We termed these top-quartile hospitals as *high-dual*, and the remaining hospitals in the bottom 3 quartiles were called *low-dual*. This definition had advantages over other definitions we considered. Other definitions included hospitals in the top quartile in (1) percentage of disproportionate patients (ie, percentage of patients on Supplemental Security Income and Medicaid^17^), (2) percentage of Medicaid days, and (3) percentage of patients with joint replacement in an area of high poverty. The definition we used better differentiated hospitals serving a high proportion of disadvantaged patients on outcomes common among disadvantaged patients with joint replacement (eg, high spending, high institutional postacute care discharge rates, and high readmission rates).^18,19^ The definition was also specific to patients with hip or knee joint replacement, whereas definitions 1 and 2 were based on all patients with hospital stays. In addition, CMS has used the proportion of dual-eligible patients as part of the value-based payment systems to identify hospitals serving disadvantaged patients.^1^ See eAppendix 2 in the Supplement for detailed information.

### Analysis 1: Changes in Spending, Health Service Use, and Quality of Care

Study outcomes included spending, health service use, and quality of care in high- and low-dual hospitals. The primary outcomes were total episode spending, discharge to institutional postacute care settings, and readmission rates within the 90-day postdischarge period. We calculated episode spending as the sum of Medicare payments for all services (except durable equipment and hospice) incurred during index hospitalization and the 90-day postdischarge period. We standardized Medicare-allowed payments to remove differences driven by wage index, indirect costs of medical education, and other special payments (eAppendix 3 in the Supplement), and reported payments in 2016 dollars.^20,21^ Discharge to institutional postacute care settings included discharges to a long-term care hospital, inpatient rehabilitation facility, skilled nursing facility, or swing bed. Previous studies have shown that discharges to institutional postacute care settings were reduced under the CJR model.^9,12,22^ Readmission rates included only relevant readmissions according to CMS definition. The CMS provided a list of 336 irrelevant conditions, and participating CJR hospitals were not responsible for readmissions associated with these conditions.^4^

We assessed the following secondary outcomes: spending at each care setting, discharge to home and home health care, and days for institutional postacute care and index hospitalization stays. We measured quality of care through complication rates,^23^ emergency department visits, mortality rates, and discharges to a skilled nursing facility with a 4- or 5-star rating (indicator of a better-quality skilled nursing facility).^24^ We also assessed the receipt of physical therapy care within 0 to 2 days of hospital discharge.^25^ eTable 1 in the Supplement provides details of each outcome.

### Analyses 2 and 3: Reconciliation Payments and Spending Reductions for a Bonus

We examined reconciliation (bonus or penalty) payments between high- and low-dual hospitals in 2016 and 2017. We grouped hospitals into 3 categories of reconciliation payments (positive, zero, and negative), and we assessed the proportion of high- and low-dual hospitals in each category.

We also estimated the mean reduction in spending that high- and low-dual hospitals would be required to achieve per episode so as to receive a bonus (ie, avoid a penalty) under the CJR model, and then we plotted the required reductions over time for high- and low-dual hospitals. We calculated this required reduction as the difference between a hospital’s historical spending per episode in 2012 to 2015 and the estimated CJR spending benchmark for the hospital. Each hospital’s benchmark in 2016 to 2018 was a weighted mean of regional historical spending and the hospital’s historical spending, transitioning to a 100% regional benchmark in 2019 and 2020. (The weight for regional spending was one-third in 2016 to 2017, two-thirds in 2018, and 1 in 2019 to 2020.) We calculated the differences using actual historical hospital spending and CJR benchmarks for the first 2 years of the CJR model. Because data on CJR benchmarks for 2018 to 2020 were not yet available, we estimated each hospital’s CJR benchmark for 2018 to 2020 by using the historical hospital and regional spending used to set benchmarks for years 1 to 2 but applying different weights to them. Additional information is provided in eAppendix 6 in the Supplement.

### Statistical Analysis

We used a difference-in-differences-in-differences, or triple-difference, approach to assess (1) the changes in outcomes associated with the CJR model in high- and low-dual hospitals and (2) the differential changes between high- and low-dual hospitals (eAppendix 4 in the Supplement). For all analyses, we used a linear ordinary least squares model at the episode level that included the index hospitalization and 90-day postdischarge period.

The key variable in the model was the 3-way interaction of 3 indicators: (1) treatment MSA (vs control MSA), (2) high-dual hospital (vs low-dual hospital), and (3) post-CJR period (vs pre-CJR period). The coefficient of the 3-way interaction measured differential changes under the CJR model between high- and low-dual hospitals. Another key variable was an interaction between treatment MSA and post-CJR period indicators. The coefficient of this interaction measured changes in low-dual hospitals associated with the CJR model. We also included interactions between the high-dual hospital indicator and year indicators of joint replacements, hospital fixed effects to account for time-invariant hospital characteristics, and year fixed effects to adjust for a secular time trend. We did not include the treatment MSA, high-dual hospital, and post-CJR period indicators separately because the first 2 indicators were perfectly collinear with hospital fixed effects, and the third indicator was perfectly collinear with year fixed effects.

In addition, we adjusted for types of joint replacement (elective knee, elective hip, and hip fracture), occurrence of major complication or comorbidity (MCC) during the hospital stay, patient age and sex, and quarter indicators. eTable 1 in the Supplement includes details of explanatory variables. We did not adjust for each patient’s race/ethnicity, Medicaid enrollment status, or preexisting medical complexity because such factors are highly correlated with high-dual hospital status. We clustered SEs on MSAs to account for correlation in error terms within MSAs. Sample weights were also included in regressions to correct for any bias caused by stratified sampling (eAppendix 1 in the Supplement). No substantial differences in adjusted outcomes existed between treatment and control MSAs across high- and low-dual hospitals during the pre-CJR period (see eTable 2 and eAppendix 5 in the Supplement), supporting the assumption of parallel trends in outcomes in the pre-CJR intervention period.^26^ We considered 2-tailed *P* < .05 to be statistically significant. Analyses were performed using R, version 3.6.0 (R Project for Statistical Computing) and Stata, version 16.0 (StataCorp LLC).

For the sensitivity analyses, we repeated the main analysis (1) using 3 other definitions of hospitals serving disadvantaged patients and (2) using 75 treatment and 121 control MSAs based on previous studies’ intention-to-treat approach.

## Results

### Characteristics of High-Dual and Low-Dual Hospitals

In total, 1165 hospitals (291 high-dual and 874 low-dual) and 768 224 patients with hip and knee joint replacement (494 013 [64.3%] women; mean [SD] age of 76 [7] years) were included. Compared with low-dual hospitals, high-dual hospitals had a higher proportion of patients with a medically complex condition (mean [SD], 10.9% [5.9%] vs 16.0% [7.9%]; *P* < .001), who were nonwhite (7.5% [7.5%] vs 21.1% [20.3%]; *P* < .001), and who had a hip fracture (18.2% [12.9%] vs 29.4% [16.4%]; *P* < .001) (Table 1). Among high-dual hospitals, 485 (55.6%) were low-volume hospitals (11-63 joint replacements each year; eTable 1 in the Supplement), compared with 612 (23.3%) among low-dual hospitals (*P* < .001). High-dual hospitals were more likely than low-dual hospitals to have lower operating margins (mean [SD], –2.3% [20.5%] vs 4.8% [13.3%]; *P* < .001). These differences were consistent after accounting for the CJR model’s 2 risk-adjustment components: percentage of patients with hip fracture or MCC (eTable 3 in the Supplement).

**Table 1.** Unadjusted Characteristics of Hospitals with High or Low Percentage of Dual-Eligible Patients Before the Comprehensive Care for Joint Replacement Model, 2012-2014

Variable	Hospital Category^a^		*P* Value^b^
	High-Dual (n = 291)	Low-Dual (n = 874)	
**Patient Characteristics, Mean (SD), %**			
Medicaid enrolled	19.0 (15.0)	4.5 (3.1)	<.001
Medically complex^c^	16.0 (7.9)	10.9 (5.9)	<.001
Nonwhite	21.1 (20.3)	7.5 (7.5)	<.001
Female	68 (7.5)	64.9 (6.0)	<.001
Age category, y			
66-70	24.5 (9.0)	27.2 (7.5)	<.001
71-75	23.2 (7.3)	25.4 (5.9)	<.001
76-80	20.2 (6.1)	20.7 (4.8)	.01
≥81	32.1 (11.6)	26.7 (9.8)	<.001
Type of joint replacement			
Elective knee	48.9 (14.4)	55.1 (12.2)	<.001
Elective hip	21.7 (9.3)	26.7 (9.4)	<.001
Hip fracture	29.4 (16.4)	18.2 (12.9)	<.001
MS-DRG			
469: Hip or knee replacement with MCC	9.9 (7.1)	6.5 (4.7)	<.001
470: Hip or knee replacement without MCC	90.1 (7.1)	93.5 (4.7)	<.001
**Hospital Characteristics, No. (%)**			
Volume of Medicare joint replacements			
Low (11-63)	485 (55.6)	612 (23.3)	<.001
Medium (64-149)	288 (33.0)	899 (34.3)	
High (150-1462)	100 (11.5)	1111 (42.4)	
Major teaching hospital	194 (22.2)	444 (16.9)	<.001
Ownership type			
For-profit	152 (17.4)	538 (20.5)	<.001
Nonprofit	573 (65.6)	1757 (67)	
Public	146 (16.7)	281 (10.7)	
Others	2 (0.2)	46 (1.8)	
Operating margin, %^d^	−2.3 (20.5)	4.8 (13.3)	<.001

Abbreviation: MCC, major complication or comorbidity; MS-DRG, Medicare Severity-Diagnosis Related Group.

^a^ High-dual hospitals are those with a high percentage of dual-eligible patients; low-dual hospitals are those with a low percentage. Dual-eligible patients are those enrolled in both Medicare and Medicaid.

^b^ *P* values were calculated with unpaired, 2-tailed *t* tests for continuous variables and χ^2^ tests for categorical variables.

^c^ Medically complex patients are defined as those with baseline Elixhauser index scores in the top decile.

^d^ Operating margin is defined as the ratio of patient care–related income to patient care–related revenue.

### Analysis 1

**Figure 1.**
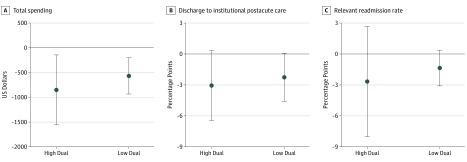
Changes in Total Spending, Discharge to Postacute Care, and Relevant Readmission Associated With the Comprehensive Care for Joint Replacement Model, 2016 (Year 1) to 2017 (Year 2) The bars indicate 95% CIs.

Figure 1 and Table 2 show changes in primary outcomes under the CJR model between high- and low-dual hospitals. Under the CJR model, total episode spending declined among both types of hospitals, decreasing by $851 (95% CI, –$1556 to –$146; *P* = .02) for high-dual hospitals and by $567 (95% CI, –$933 to –$202; *P* = .003) for low-dual hospitals. However, the size of these changes was not statistically significantly different between high- and low-dual hospitals (difference, –$284; 95% CI, –$981 to $413; *P* = .42). The CJR model was not associated with changes in 2 other primary outcomes (ie, discharge to institutional postacute care and readmission rates) among high- and low-dual hospitals.

**Table 2.** Adjusted Changes in Spending, Health Service Use, and Quality of Care Associated With the CJR Model, 2016-2017^a^

Variable	Hospital Category^b^				High-Dual vs Low-Dual	
	High-Dual		Low-Dual			
	Changes in Outcomes Under CJR (95% CI)	*P* Value	Changes in Outcomes Under CJR (95% CI)	*P* Value	Differences (95% CI)	*P* Value
Total No. of hospitals	291	NA	874	NA	NA	NA
Total No. of replacements	109 649	NA	726 378	NA	NA	NA
Primary outcomes^c^						
Total spending, US $	−851 (−1556 to −146)	.02	−567 (−933 to −202)	.003	−284 (−981 to 413)	.42
Discharge to institutional postacute care, %	−0.03 (−0.06 to 0.003)	.08	−0.02 (−0.05 to 0.001)	.06	−0.01 (−0.04 to 0.03)	.65
Relevant readmission rates, %	−0.03 (−0.08 to 0.03)	.33	−0.01 (−0.03 to 0.004)	.13	−0.01 (−0.06 to 0.03)	.56
Spending, US $						
Index hospitalization	−88 (−214 to 39)	.17	−33 (−83 to 17)	.19	−55 (−189 to 79)	.42
Relevant readmission	−141 (−335 to 52)	.15	15 (−48 to 79)	.63	−157 (−360 to 46)	.13
Institutional postacute care	−750 (−1419 to −81)	.03	−525 (−862 to −187)	.002	−225 (−782 to 332)	.43
Long-term care hospital	26 (−65 to 117)	.58	17 (−26 to 60)	.42	8 (−72 to 89)	.84
Inpatient rehabilitation facility	−241 (−688 to 207)	.29	−200 (−419 to 19)	.07	−41 (−444 to 363)	.84
Skilled nursing facility	−456 (−920 to 9)	.05	−324 (−581 to −68)	.01	−132 (−544 to 281)	.53
Home health agency	133 (−119 to 385)	.30	−15 (−178 to 148)	.86	148 (−42 to 338)	.13
Swing bed	−79 (−176 to 19)	.11	−17 (−71 to 36)	.52	−61 (−173 to 50)	.28
Outpatient facility	−22 (−68 to 25)	.36	25 (0 to 50)	.05	−47 (−95 to 1)	.06
Professional service	16 (−153 to 186)	.85	−36 (−108 to 37)	.34	52 (−121 to 225)	.55
Health service use						
Discharge to home health, %	0.06 (−0.002 to 0.12)	.06	0.02 (−0.03 to 0.07)	.41	0.04 (−0.01 to 0.09)	.14
Discharge to home, %	−0.01 (−0.05 to 0.02)	.51	0 (−0.04 to 0.05)	.84	−0.02 (−0.06 to 0.03)	.44
Mean LOS, d						
Institutional postacute care facility	−1.2 (−2.3 to −0.1)	.03	−0.8 (−1.3 to −0.3)	.003	−0.4 (−1.3 to 0.5)	.37
Index hospitalization	−0.04 (−0.16 to 0.08)	.53	−0.03 (−0.10 to 0.04)	.36	−0.01 (−0.12 to 0.11)	.92
Quality of care, %						
Complication rates	−0.003 (−0.01 to 0.003)	.30	−0.0001 (−0.003 to 0.002)	.90	−0.003 (−0.01 to 0.003)	.38
ED visit rates	−0.01 (−0.02 to 0.005)	.22	0.001 (−0.004 to 0.006)	.65	−0.01 (−0.02 to 0.004)	.16
Mortality rates	−0.002 (−0.01 to 0.003)	.49	0.0001 (0 to 0.002)	.91	−0.002 (−0.01 to 0.003)	.47
Skilled nursing facility rating of 4 or 5 stars^d^	0.08 (0.01 to 0.14)	.02	0.05 (0.06 to 0.09)	.03	0.03 (−0.05 to 0.10)	.48
Timely physical therapy^e^	0.04 (−0.05 to 0.12)	.42	0.001 (−0.04 to 0.04)	.96	0.03 (−0.06 to 0.13)	.47

Abbreviations: CJR, Comprehensive Care for Joint Replacement; ED, emergency department; LOS, length of stay; NA, not applicable.

^a^ Results were based on a triple-difference approach, adjusted for types of joint replacement, occurrence of major complications or comorbidities during the hospital stay, patient age and sex, hospital fixed effects, year fixed effects, and quarter fixed effects.

^b^ High-dual hospitals are those with a high percentage of dual-eligible patients; low-dual hospitals are those with a low percentage. Dual-eligible patients are those enrolled in both Medicare and Medicaid.

^c^ eTable 1 in the Supplement provides detailed definitions of the outcome variables.

^d^ Skilled nursing facility rating of 4 or 5 stars was estimated only among patients who were discharged to skilled nursing facilities.

^e^ Timely physical therapy was estimated only among patients who received elective knee replacement and were discharged home.

Table 2 shows changes in secondary outcomes under the CJR model between the hospital groups. The model was associated with decreases in institutional postacute care spending (high-dual hospitals: –$750 [95% CI, –$1419 to –$81; *P* = .03]; low-dual hospitals: –$525 [95% CI, –$862 to –$187; *P* = .002]) and mean length of stay at institutional postacute care facilities (high-dual hospitals: –1.2 days [95% CI, –2.3 to –0.1 days; *P* = .03]; low-dual hospitals: –0.8 days [95% CI, –1.3 to –0.3 days; *P* = .003]) in both groups. None of these changes differed between high- and low-dual hospitals (institutional postacute care spending: −$225; 95% CI, −$782 to $332; *P* = .43; length of stay at institutional postacute care facilities: −0.4 days; 95% CI, −1.3 to 0.5 days; *P* = .37).

Most quality measures did not change under the CJR model in both hospital groups. The exception was discharges to a skilled nursing facility with a 4- or 5-star rating, which increased by 8 percentage points (95% CI, 1-14; *P* = .02) for high-dual hospitals and by 5 percentage points (95% CI, 6-9 percentage points; *P* = .03) for low-dual hospitals. However, the difference between the 2 groups was not statistically significant (3 percentage points; 95% CI, −5 to 10 percentage points; *P* = .48). See eTable 4 in the Supplement for the level in each outcome before and after CJR model implementation in high- and low-dual hospitals.

The results for high-dual and low-dual hospitals were qualitatively similar, with a few exceptions when we repeated the analysis using 3 alternative definitions of hospitals serving a high percentage of disadvantaged patient populations (eTables 5-7 in the Supplement). For example, 1 exception was a finding of greater decreases in discharges to institutional postacute care (by 3 percentage points) among hospitals with low disproportionate patient percentages compared with those with high disproportionate patient percentages. Using an intention-to-treat approach (75 treatment and 121 control MSAs), the results stayed almost the same, with slightly smaller effect sizes (eTable 8 in the Supplement).

### Analyses 2 and 3

**Figure 2.**
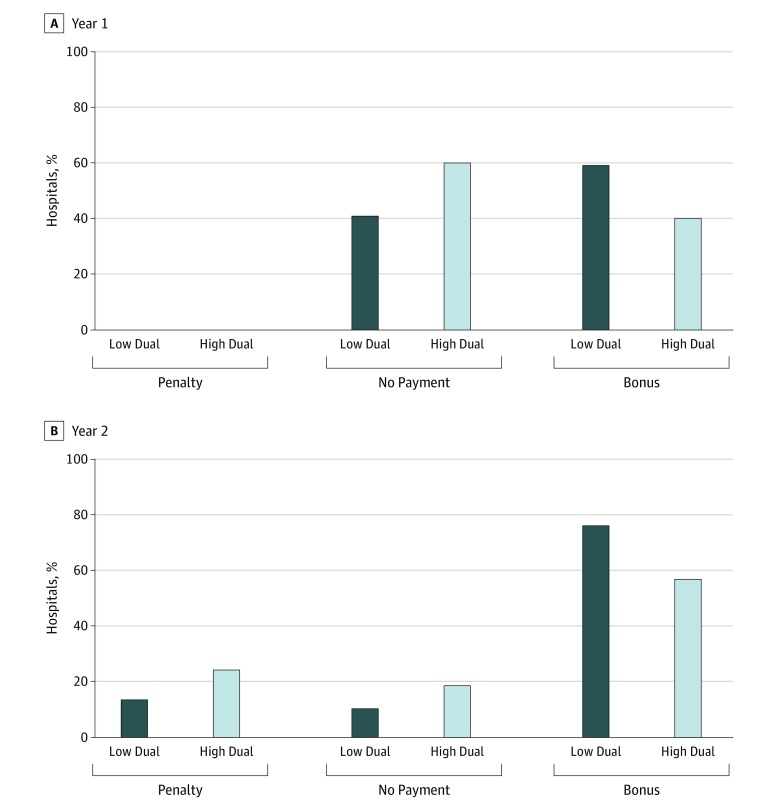
Comprehensive Care for Joint Replacement (CJR) Model Bonus and Penalty, 2016 (Year 1) to 2017 (Year 2) Hospitals with a high percentage of dual-eligible (ie, enrolled in both Medicare and Medicaid) patients (high-dual) were less likely than hospitals with a low percentage of dual-eligible patients (low-dual) to receive a CJR bonus (40.3% vs 59.1% in year 1; 56.9% vs 76.0% in year 2). In year 1, hospitals were exempt from a penalty even if their episode spending was above the benchmark. In year 2, high-dual hospitals were more likely than low-dual hospitals to be penalized (24.3% vs 13.7%). In year 2, high-dual hospitals received a mean of $1173 as a bonus if they received any, compared with a $1194 bonus for low-dual hospitals. High-dual hospitals faced $1114 as a penalty, if any, compared with the $822 penalty for low-dual hospitals.

Figure 2 shows reconciliation payments for high-dual and low-dual hospitals in the first 2 years of the CJR model. In 2016, hospitals were exempt from a penalty even if their episode spending exceeded the benchmark. High-dual hospitals were less likely than low-dual hospitals to receive a bonus (40.3% vs 59.1% in 2016 [year 1]; 56.9% vs 76.0% in 2017 [year 2]).

**Figure 3.**
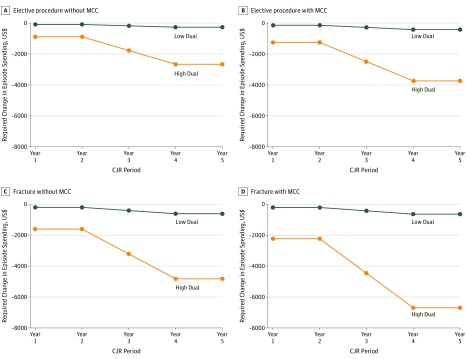
Required Change in Episode Spending for Receiving a Comprehensive Care for Joint Replacement (CJR) Bonus (or Avoiding a Penalty), Years 1 to 5 All dollar values were adjusted for wage index differentials and reported in 2016 dollars. eTable 9 in the Supplement shows the dollar values corresponding to each point in this figure, and eAppendix 6 in the Supplement explains how these values were calculated. MCC indicates major complications or comorbidities.

Figure 3 and eTable 9 in the Supplement show the differences between historical hospital spending and CJR spending benchmarks for high- and low-dual hospitals for 4 types of patients. The CJR model sets 4 separate benchmarks for the type of surgical procedure (elective vs hip fracture replacement) and the occurrence of MCCs during the hospital stay. Each line in Figure 3 represents the spending reductions necessary to obtain a bonus. In 2016 and 2017, across all 4 patient types, high-dual hospitals were required to reduce spending by $887 to $2231, whereas low-dual hospitals needed to reduce spending by only $89 to $215. This difference would grow over time with a transition to 100% regional spending benchmarks. By year 5 of the CJR model, high-dual hospitals would be required to reduce spending by $6694 per episode for a bonus for patients with hip fracture who experienced MCCs, whereas low-dual hospitals would need to reduce spending by only $644.

## Discussion

Using 100% Medicare claims for patients who received hip and knee joint replacement from 2012 to 2017, we found that both high- and low-dual hospitals decreased total spending under the CJR model, but the magnitude of decreases did not differ between the 2 groups. However, high-dual hospitals were less likely to receive a bonus. In addition, we estimated that the CJR model would require high-dual hospitals to reduce spending more substantially to obtain a bonus compared with low-dual hospitals. In 2016 and 2017, across 4 patient types, high-dual hospitals needed to reduce episode spending by $887 to $2231 for a bonus, but the corresponding required savings for low-dual hospitals were only $89 to $215. The percentage of high-dual hospitals that do not receive a bonus (or have to pay a penalty) is expected to further increase in later years of the CJR model as its spending benchmarks become based solely on regional spending.

The lower quality and higher spending among high-dual hospitals may be more closely associated with inefficient operations than with patient characteristics. In this case, the CJR spending benchmarks should appropriately incentivize high-dual hospitals to improve in these areas. However, lower performance of high-dual hospitals may also reflect patients’ complexity. If so, the way CJR spending benchmarks are currently set up may penalize already financially challenged hospitals, restrict their ability to provide a full set of services to vulnerable populations, and inhibit other worthwhile investments.

In this vein, we suggest that policy makers consider accounting for patients’ social and medical complexities when setting CJR spending benchmarks. Currently, under the CJR model, a hospital’s benchmarks are a weighted mean of historical hospital and regional spending for the first 3 years, transitioning to a benchmark based entirely on historical regional spending in 2019. To receive a bonus or avoid a penalty, hospitals with historical spending that was higher than the regional mean, such as high-dual hospitals, would need to decrease spending substantially more than hospitals in the same region with historical spending that was lower than the regional mean.^5^ However, high-dual hospitals may struggle to reduce spending given their high share of patients with social and medical complexities.

Facing similar problems, other value-based programs have modified their design in favor of high-dual hospitals. Medicare’s Hospital Readmissions Reduction Program, which was designed to reduce readmission, recently divided all participating hospitals into 5 groups by their percentage of dual-eligible discharges, using the median readmission rates within each group as a threshold for each hospital.^27^ The CJR model may be improved by incorporating separate tracks for high-dual hospitals. By competing with others within their own track, high-dual hospitals would face incentives to improve without incurring unnecessary penalties for serving disadvantaged patients.^28^

Multiple studies have found that hospitals serving a high percentage of disadvantaged populations were more likely to get penalized under the CJR model.^5,29,30^ However, the present study went further in assessing changes in spending, health services, and quality of care in those hospitals under the CJR model. We also analyzed the spending reductions required for a CJR bonus for high- and low-dual hospitals, providing practical policy implications for CJR spending benchmarks.

As the CJR model evolved to become a voluntary model in 33 of the original 67 MSAs in 2018, many hospitals left the program, with high-dual hospitals disproportionately represented in that group.^31^ Going forward, we believe that policy makers must find ways for high-dual hospitals to be included in value-based payment efforts as they develop and implement models designed to improve care and slow the growth of health care spending.

### Limitations

This study has several limitations. First, we excluded durable medical equipment and hospice spending from total spending. However, these costs accounted for less than 1% of total spending, and other studies have suggested that the CJR model was not associated with changes in these 2 types of spending.^11^ Second, we excluded patients with joint replacement who were younger than 66 years. Third, we did not assess patient selection between high- and low-dual hospitals. High-dual hospitals may end up serving even greater percentages of high-risk patients under the CJR model if other hospitals begin sending high-risk patients to high-dual hospitals.^32,33,34^

## Conclusions

This study found that, under the CJR model, high-dual and low-dual hospitals have both reduced their spending, and the size of reduction did not differ between the 2 groups of hospitals. However, high-dual hospitals were less likely to receive a bonus. In the foreseeable future, high-dual hospitals would be required to make more substantial cuts to receive a bonus or avoid a penalty.

## Notes

Supplement. **eFigure 1.** MSA Selection Process for CJR Participation **eFigure 2.** Map of Treatment and Control MSAs by Historical Spending of Care Episode **eFigure 3.** Study Sample Selection Process **eAppendix 1.** MSA Randomization for CJR Participation and Calculation of Sampling Weights **eAppendix 2.** Measure of Hospitals Serving a High Percentage of Disadvantaged Joint Replacement Patient Population **eAppendix 3.** Standardization of Medicare-Allowed Payments **eAppendix 4.** Difference-in-Differences-in-Differences or Triple-Difference Approach **eAppendix 5.** Comparison of Adjusted Outcomes Between Treatment and Control MSAs Across High- and Low-Dual Hospitals Each Year in the Pre-CJR Period **eAppendix 6.** Required Changes in Episode Spending Each Hospital Had/Has to Receive a Bonus (or Avoid a Penalty) in CJR Years 1-5 **eTable 1.** Definition and Data Source for Outcomes and Explanatory Variables **eTable 2.** Adjusted Outcomes Between Treatment and Control MSAs Among Low-Dual Hospitals and High-Dual Hospitals Each Year in the Pre-CJR Period (2012-2014) **eTable 3.** Adjusted Hospital-Level Characteristics of High-Dual and Low-Dual Hospitals in the Pre-CJR Period (2012-2014) **eTable 4.** Differential Changes in Outcomes Before and After Implementation of the CJR Model Among Low-Dual Hospitals and High-Dual Hospitals **eTable 5.** Adjusted Changes in Outcomes Associated With CJR Between Hospitals in the Top and Bottom 3 Quartiles in Disproportionate Patient Percentages (DPP) in CJR Years 1-2 (April 2016 - Dec 2017) **eTable 6.** Adjusted Changes in Outcomes Associated With CJR Between Hospitals in the Top and Bottom 3 Quartiles in Medicaid Day Percentages in CJR Years 1-2 (April 2016 - Dec 2017) **eTable 7.** Adjusted Changes in Outcomes Associated With CJR Between Hospitals in the Top and Bottom 3 Quartiles in Percentage of Patients in an Area of High Poverty in CJR Years 1-2 (April 2016 - Dec 2017) **eTable 8.** Intention-to-Treat Approach: Adjusted Changes in Spending, Health Service Use, and Quality of Care Associated With the CJR Model Between High-Dual and Low-Dual Hospitals in CJR Years 1-2 (April 2016 - Dec 2017) **eTable 9.** Required Change in Episode Spending Each Hospital Had/Has to Receive a Bonus (or Avoid a Penalty) in CJR Years 1-5
