# Supplementary material for: Association of the Mandatory Medicare Bundled Payment With Joint Replacement Outcomes in Hospitals With Disadvantaged Patients
Source: JAMA Netw Open. 2019 Nov 6;2(11):e1914696. doi: 10.1001/jamanetworkopen.2019.14696 (PMC6865278; doi:10.1001/jamanetworkopen.2019.14696)
Supplement: Supplement. — eFigure 1. MSA Selection Process for CJR Participation eFigure 2. Map of Treatment and Control MSAs by Historical Spending of Care Episode eFigure 3. Study Sample Selection Process eAppendix 1. MSA Randomization for CJR Participation and Calculation of Sampling Weights eAppendix 2. Measure of Hospitals Serving a High Percentage of Disadvantaged Joint Replacement Patient Population eAppendix 3. Standardization of Medicare-Allowed Payments eAppendix 4. Difference-in-Differences-in-Differences or Triple-Difference Approach eAppendix 5. Comparison of Adjusted Outcomes Between Treatment and Control MSAs Across High- and Low-Dual Hospitals Each Year in the Pre-CJR Period eAppendix 6. Required Changes in Episode Spending Each Hospital Had/Has to Receive a Bonus (or Avoid a Penalty) in CJR Years 1-5 eTable 1. Definition and Data Source for Outcomes and Explanatory Variables eTable 2. Adjusted Outcomes Between Treatment and Control MSAs Among Low-Dual Hospitals and High-Dual Hospitals Each Year in the Pre-CJR Period (2012-2014) eTable 3. Adjusted Hospital-Level Characteristics of High-Dual and Low-Dual Hospitals in the Pre-CJR Period (2012-2014) eTable 4. Differential Changes in Outcomes Before and After Implementation of the CJR Model Among Low-Dual Hospitals and High-Dual Hospitals eTable 5. Adjusted Changes in Outcomes Associated With CJR Between Hospitals in the Top and Bottom 3 Quartiles in Disproportionate Patient Percentages (DPP) in CJR Years 1-2 (April 2016 - Dec 2017) eTable 6. Adjusted Changes in Outcomes Associated With CJR Between Hospitals in the Top and Bottom 3 Quartiles in Medicaid Day Percentages in CJR Years 1-2 (April 2016 - Dec 2017) eTable 7. Adjusted Changes in Outcomes Associated With CJR Between Hospitals in the Top and Bottom 3 Quartiles in Percentage of Patients in an Area of High Poverty in CJR Years 1-2 (April 2016 - Dec 2017) eTable 8. Intention-to-Treat Approach: Adjusted Changes in Spending, Health Service Use, and Quality of Care Associated W [file jamanetwopen-2-e1914696-s001.pdf]

Supplementary Online Content

Kim H, Meath THA, Dobbertin K, Quiñones AR, Ibrahim SA, McConnell KJ. Association of the mandatory Medicare bundled payment with joint replacement outcomes in hospitals with disadvantaged patients. *JAMA Netw Open*. 2019;2(11):e1914696. doi:10.1001/jamanetworkopen.2019.14696

eFigure 1. MSA Selection Process for CJR Participation
eFigure 2. Map of Treatment and Control MSAs by Historical Spending of Care Episode
eFigure 3. Study Sample Selection Process
eAppendix 1. MSA Randomization for CJR Participation and Calculation of Sampling Weights
eAppendix 2. Measure of Hospitals Serving a High Percentage of Disadvantaged Joint Replacement Patient Population
eAppendix3. Standardization of Medicare-Allowed Payments
eAppendix 4. Difference-in-Differences-in-Differences or Triple-Difference Approach
eAppendix 5. Comparison of Adjusted Outcomes Between Treatment and Control MSAs Across High- and Low-Dual Hospitals Each Year in the Pre-CJR Period
eAppendix 6. Required Changes in Episode Spending Each Hospital Had/Has to Receive a Bonus (or Avoid a Penalty) in CJR Years 1-5
eTable 1. Definition and Data Source for Outcomes and Explanatory Variables
eTable 2. Adjusted Outcomes Between Treatment and Control MSAs Among Low-Dual Hospitals and High-Dual Hospitals Each Year in the Pre-CJR Period (2012-2014)
eTable 3. Adjusted Hospital-Level Characteristics of High-Dual and Low-Dual Hospitals in the Pre-CJR Period (2012-2014)
eTable 4. Differential Changes in Outcomes Before and After Implementation of the CJR Model Among Low-Dual Hospitals and High-Dual Hospitals
eTable 5. Adjusted Changes in Outcomes Associated With CJR Between Hospitals in the Top and Bottom 3 Quartiles in Disproportionate Patient Percentages (DPP) in CJR Years 1-2 (April 2016 - Dec 2017)
eTable 6. Adjusted Changes in Outcomes Associated With CJR Between Hospitals in the Top and Bottom 3 Quartiles in Medicaid Day Percentages in CJR Years 1-2 (April 2016 - Dec 2017)
eTable 7. Adjusted Changes in Outcomes Associated With CJR Between Hospitals in the Top and Bottom 3 Quartiles in Percentage of Patients in an Area of High Poverty in CJR Years 1-2 (April 2016 - Dec 2017)
eTable 8. Intention-to-Treat Approach: Adjusted Changes in Spending, Health Service Use, and Quality of Care Associated With the CJR Model Between High-Dual and Low-Dual Hospitals in CJR Years 1-2 (April 2016 - Dec 2017)
eTable 9. Required Change in Episode Spending Each Hospital Had/Has to Receive a Bonus (or Avoid a Penalty) in CJR Years 1-5

This supplementary material has been provided by the authors to give readers additional information about their work.

eFigure 1. MSA Selection Process for CJR Participation

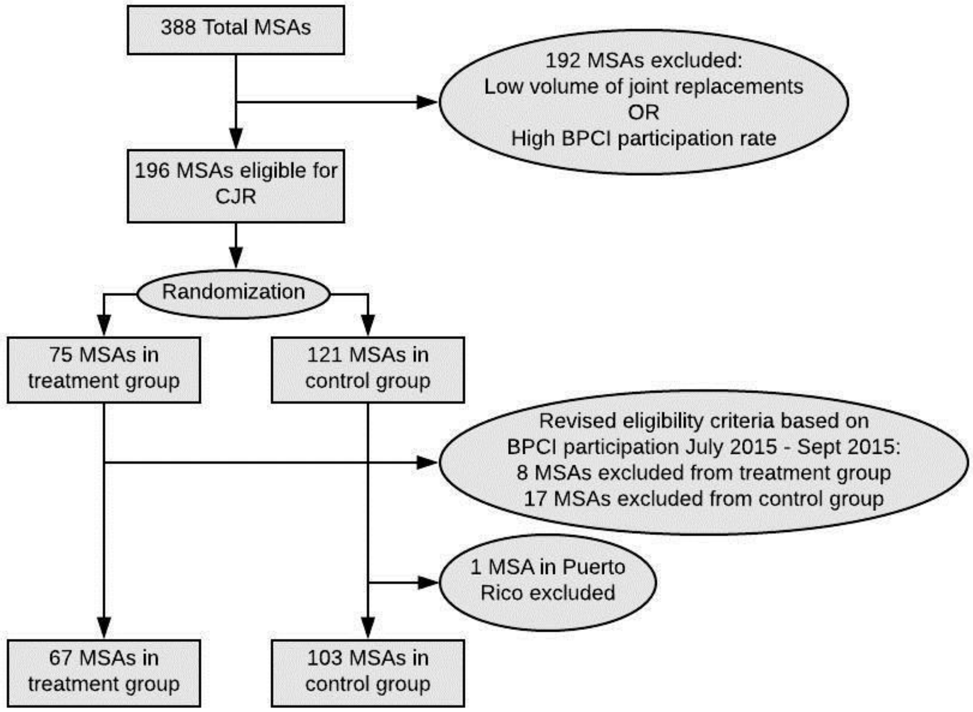

Notes: MSA is metropolitan statistical area, BPCI is Bundled Payment for Care Improvement Initiative, and CJR is Comprehensive Care for Joint Replacement model

© 2019 Kim H et al. *JAMA Network Open*

eFigure 2. Map of Treatment and Control MSAs by Historical Spending of Care Episode

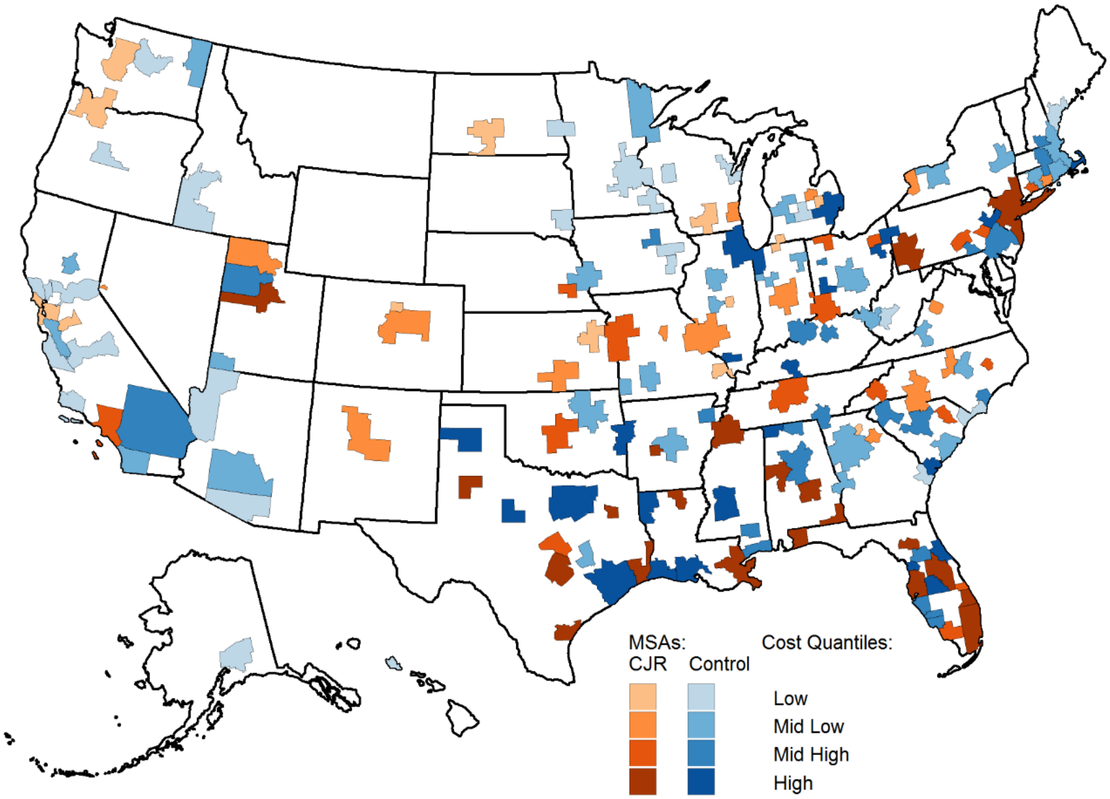

Notes: MSA is metropolitan statistical area; CJR model is Comprehensive Care for Joint Replacement model.

© 2019 Kim H et al. *JAMA Network Open*

eFigure 3. Study Sample Selection Process

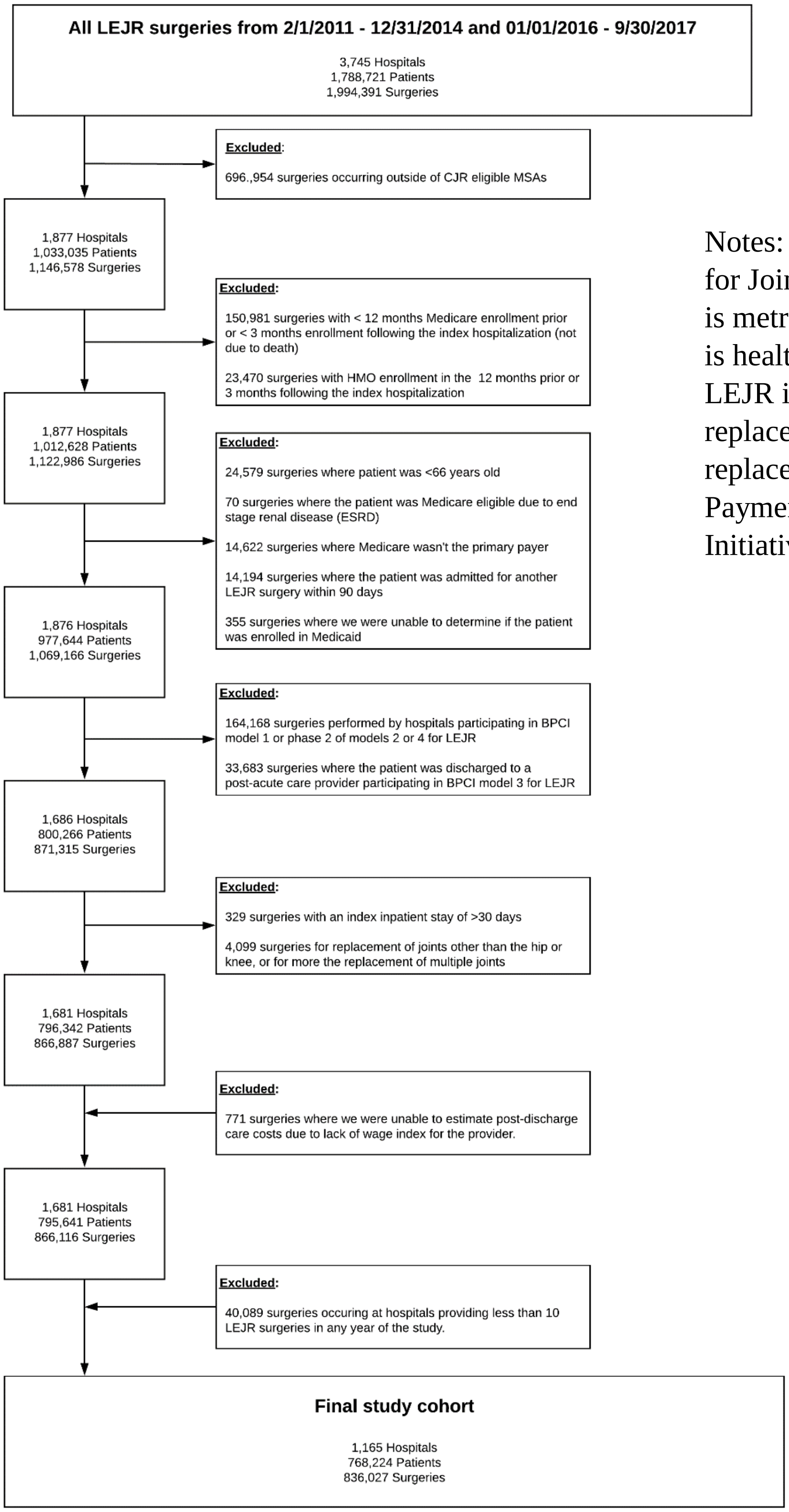

Notes: CJR is Comprehensive Care for Joint Replacement Model. MSA is metropolitan statistical area. HMO is health maintenance organization. LEJR is lower extremity joint replacements (i.e., hip and knee joint replacements). BCPI is Bundled Payments for Care Improvement Initiative.

© 2019 Kim H et al. *JAMA Network Open*

eAppendix 1. MSA Randomization for CJR Participation and Calculation of Sampling Weights

CMS used a stratified clustered random sampling, with metropolitan statistical areas (MSAs) serving as the clusters, to assign MSAs for CJR participation (see eFigure 1). CJR participation was mandatory for all hospitals within a selected MSA.

1. From the 388 MSAs in the United States, CMS excluded 192 MSAs from CJR model participation due to their low hip and knee replacement volume or high rates of participation in the Bundled Payments for Care Improvement (BPCI).
2. The remaining 196 eligible MSAs were grouped into 8 strata based on their population size and historical care episode spending. CMS identified cut-points for strata using K-means factor analysis.
3. CMS then randomly selected MSAs for CJR participation within each stratum, with a higher sampling probability (oversampling) in the strata with the higher historical spending. They implemented random sampling using the "PROC SURVEYSELECT" statement in SAS. As a result of the random selection, CMS announced 75 treatment MSAs for CJR participation and 121 control MSAs. Additional information about the randomization process is available in the CJR Final Rule (Section III A).[1] CJR participation was mandatory for hospitals located in the 75 selected MSAs unless they were already participating in the BPCI model one, or phase two of model two or model four.
4. Under this sampling approach (described in the step 1-3), it would be possible to calculate sampling weights using the selection probability applied to each of 8 strata.
5. However, CMS retroactively changed the eligibility criteria for MSAs after the initial randomization described in the step 3. This led to the exclusion of 8 treatment MSAs that had been selected for the CJR participation and 17 control MSAs that were not selected for CJR but would have been excluded under this new criteria. Additionally, we excluded the San Juan, Puerto Rico MSA where the healthcare system was struck by Hurricane Maria in 2017. As a result of further exclusion of those 26 (=8+17+1) MSAs, we updated the selection probability for each MSA strata conditional on the new eligibility criteria:

$$P(CJR_{strata} = 1 \mid Eligibility_{strata} = 1) = \frac{Number\ of\ MSAs\ selected\ from\ strata\ after\ exclusion}{Number\ of\ MSAs\ eligible\ in\ strata\ after\ exclusion} \quad (1)$$

We then calculated updated raw selection and non-selection weights from these probabilities using equation (2).

$$w = \begin{cases} 1/p & if\ selected = 1 \\ 1/(1-p) & if\ selected = 0 \end{cases} \quad (2)$$

Finally, we normalized the selection weights for each strata by dividing them by the sum of the raw strata-level selection weights, and normalize the non-selection weights by dividing them by the sum of the raw strata-level non-selection weights.

© 2019 Kim H et al. *JAMA Network Open*

eAppendix 2. Measure of Hospitals Serving a High Percentage of Disadvantaged Joint Replacement Patient Population

We considered four measures to identify hospitals serving a high percentage of disadvantaged patient populations: (1) hospitals with a high proportion of hip and knee joint replacement patients who were dually eligible for both Medicaid and Medicare, (2) hospitals with a high disproportionate patient percentage (DPP), (3) hospitals with a high proportion of inpatient days attributable to patients eligible for Medicaid but not Medicare (Medicaid ratio) and (4) hospitals with a high proportion of hip and knee joint replacement patients living in areas of high poverty. Please note that DPP-based definition and Medicaid-ratio-based definition are measures of low-income patients served for all inpatient services while the other two definitions are specific to hip and knee joint replacement patients.

<u>Measure 1 - Hospitals with a high proportion of hip and knee joint replacement patients dually eligible for Medicaid and Medicare</u>
Medicaid eligibility for hip and knee joint replacement patients was obtained from the Medicare Master Beneficiary Summary File. Patients were considered dually eligible only if they were enrolled in full Medicaid coverage for the month of their discharge (defined as values of ’02’, ‘04’, or ‘08’ in the DUAL_STUS_CD_<MM> field of the Master Beneficiary Summary File).[2]

<u>Measure 2 - Hospitals with a high DPP</u>
DPP is a hospital characteristic used by CMS to identify hospitals that serve a disproportionately high percentage of low-income patients. These hospitals are eligible for additional payments from CMS. DPP is calculated as the sum of the proportion of Medicare inpatient days attributable to patients covered under both Medicare part A and Supplemental Social Security Income and the proportion of all inpatient days that are attributable to patients eligible for Medicaid but not Medicare part A (See Equation (1)).

$$\mathrm{DPP} = \frac{\text{Inpatient Days}_{\text{Medicare+SSI}}}{\text{Inpatient Days}_{\text{Medicare}}} + \frac{\text{Inpatient Days}_{\text{Medicaid}}}{\text{Inpatient Days}_{\text{all}}} \quad (1)$$

<u>Measure 3 - Hospitals with a high proportion of inpatient days attributable to patients eligible for Medicaid but not Medicare.</u>
The proportion of inpatient days attributable to patients eligible for Medicaid but not Medicare part A, also called the Medicaid ratio, is one component of the DPP calculation used by CMS to identify hospitals eligible for additional payments.

$$\mathrm{MedicaidRatio} = \frac{\text{Inpatient Days}_{\text{Medicaid}}}{\text{Inpatient Days}_{\text{all}}} \quad (2)$$

<u>Measure 4 - Hospitals with a high proportion of high and knee joint replacement patients living in areas of high poverty</u>
Patients were considered living in high poverty areas if they lived in a ZIP code where at least 20% of the residents over the age of 64 reported living under the federal poverty level. ZIP code level poverty rates were obtained from American Community Survey (ACS) 5-year estimates from 2013-2017 (ACS Table S1701).

© 2019 Kim H et al. *JAMA Network Open*

Comparing Measures 1 to 4

Our goal was to identify which measure was best able to differentiate hospitals serving a high proportion of disadvantaged patients from other hospitals on outcomes common among disadvantaged joint replacement patients (e.g., high spending, high rates of institutional post-acute care discharge, and high readmission rates).[6,7] For each measure, we chose a threshold, and then compared hospitals above that threshold with hospitals below that threshold on three outcomes (spending, discharge to institutional post-acute care, and readmission). We then repeated this process over a range of possible thresholds.

For example, for the DPP measure, we compared hospitals in the top 10% of DPP to those in the bottom 90%, then compared those in the top 15% to those in the bottom 85%, then those in the top 20% to those in the bottom 80%, and so on. By plotting outcomes across the continuum of possible thresholds, we were able to visually assess which measure and threshold maximized differentiation between two groups of hospitals while avoiding small group sizes. Once we chose a measure and threshold, we defined hospitals above the threshold as hospitals serving a high proportion of disadvantaged patients. We used the pre-CJR data for these calculations because the implementation of CJR may influence patient composition of each hospital.

As seen in Figure A below, the dual-eligible based definition – one using the proportion of dual-eligible hip and knee joint replacement patients - provided better differentiation in outcomes than the three other definitions. Within this measure, differentiation was relatively stable across thresholds, and therefore we chose to define hospitals serving a high percentage of disadvantaged patient populations as those in the top 25% for proportion dual-eligible hip and knee joint replacement patients for both simplicity and to ensure there was a sufficient number of hospitals in each group.

CMS has also used the proportion of dual-eligibles as part of their value-based payment systems to identify hospitals serving disadvantaged patient populations after the dual-eligibility status was found to be the most powerful predictor of poor outcomes under federal payment systems.[3] One example is the Hospital Readmission Reduction Program, where target readmission rates for each hospital are set within strata based on the proportion of dual-eligible discharges.[1]
We also preferred the dual-eligible-based measure because it was specific to hip and knee joint replacement patients. In contrast, DPP-based and Medicaid-ratio-based measures are calculated based on all patients with inpatient stays (regardless of their receipt of joint replacement surgeries).

© 2019 Kim H et al. *JAMA Network Open*

Figure A. Spending, discharge to institutional post-acute care, and readmission rates between hospitals above and below each quantile threshold for Measure 1-4.

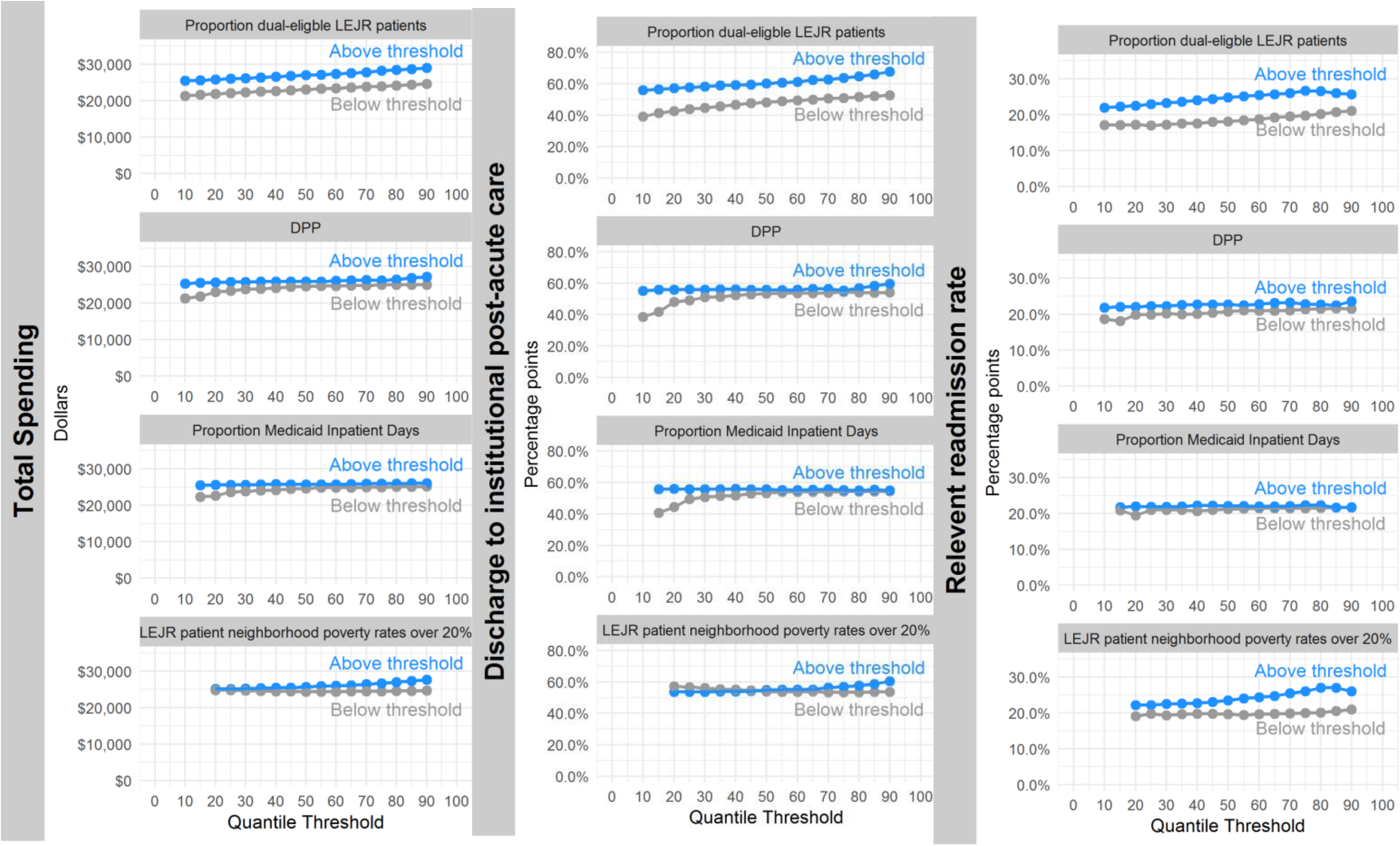

Notes: LEJR is lower extremity joint replacement (i.e., hip and knee joint replacement); DPP is disproportionate patient percentage. The “above threshold” line represents the mean outcome among hospitals above the quantile threshold while the “below threshold” line is the mean outcome among hospitals below that threshold. We defined hospitals above the selected threshold of the selected measure as those serving a high proportion of disadvantaged patient populations.

© 2019 Kim H et al. *JAMA Network Open*

eAppendix 3. Standardization of Medicare-Allowed Payments

Based on Gottlieb et al.[4] with some modifications, we standardized all Medicare-allowed payments to remove payment differences driven by wage index, indirect costs of medical education, and other special payments by taking the following three steps:

(1) Calculate provider wage index We calculated provider wage index using MSA wage index.[5] CMS annually update MSA wage index based on the cost of living across MSAs. The calculation of provider wage index for outpatient claims was different because the portion of the claim payment amount affected by MSA wage index was lower for outpatient claims (0.60) than it was for all other claim types (0.75).

$$Provider\ wage\ index\ for\ outpatient\ claims = 0.40 + (0.60\ \times\ MSA\ wage\ index)$$
$$Provider\ wage\ index\ for\ all\ other\ claims = 0.25 + (0.75\ \times\ MSA\ wage\ index)$$

(2) Standardize Medicare-allowed payments We standardized payments in index hospitalization claims and 90-day readmission claims differently from payments in carrier, outpatient, and post-acute care claims (i.e., skilled nursing facility, inpatient rehab, long-term care hospital, swing bed, and home health). Medicare-allowed payments for hospitals differed based on not only wage index but also teaching hospital status, safety-net hospital status, and others.

a. Standardization of payments in index inpatient and readmission claims

$$Standardized\ payment\ amount = National\ DRG\ price\ \times DRG\ weight + \frac{Outlier\ payment\ amount}{Provider\ wage\ index}$$

b. Standardization of payments in carrier, outpatient, and post-acute care claims

$$Standardized\ payment\ amount = \frac{Actual\ payment\ amont}{Provider\ wage\ index}$$

(3) Normalize standardized Medicare-allowed payments We multiplied standardized payment amounts with λ to make aggregated standardized payment amounts equal to aggregated Medicare-allowed payment amounts each year.

$$\lambda = \frac{Total\ actual\ payment\ amount\ by\ year\ and\ claim\ type}{Total\ standardized\ payment\ amount\ by\ year\ and\ claim\ type}$$

$$Normalized, standardized\ payment\ amount = \lambda \times \text{Standardized payment amount}$$

© 2019 Kim H et al. *JAMA Network Open*

eAppendix 4. Difference-in-Differences-in-Differences or Triple-Difference Approach

The triple-difference approach measures the difference between two estimates derived through the difference-in-differences (DD) approach. In our case, we considered one DD estimate for high-dual hospitals and the other for low-dual hospitals. The DD estimate for high-dual hospitals is the average difference (occurring with the CJR implementation) in their outcomes in treatment MSAs subtracted by the average differences in the control MSAs. Likewise, the DD estimate for low-dual hospitals measures changes in outcomes under CJR model among low-dual hospitals. The difference between the DD estimates of high- and low-dual measures the differential changes between two groups of hospitals under CJR. More specifically, we estimated the following care-episode-level regression:

$$Y_{ihmt} = \beta_0 + \beta_1 Treat_m \times Post_t \times HighDual_{ihm} + \beta_2 Treat_m \times Post_t + \sum_t \beta_{3t} Year_t \times HighDual_{ihm} + XB + \sum_h \phi_h Hospital_h + \sum_t \gamma_t Year_t + \sum_t \theta_t Quarter_t + \varepsilon_{ihmt}$$

where Y_{ihmt} is an outcome variable for joint replacement i that occurred in hospital h located in MSA m in year t. $Treat_m$ takes a value of 1 if a joint replacement occurred in the treatment MSAs and 0 otherwise. $Post_t$ takes a value of 1 if an observation occurred in 2016-2017 and 0 otherwise. $HighDual_{ihm}$ takes a value of 1 for high-dual hospitals and 0 for low-dual hospitals. X includes types of joint replacement, occurrence of major complications or comorbidities during the hospital stay, and patient age and gender. $Hospital_h$, $Year_t$, and $Quarter_t$ represent hospital, year, and quarter fixed effects. β_1 is the estimate of interest and measures the differential changes in outcomes between high- and low-dual hospitals under CJR. β_2 is another estimate of interest and measures changes in outcomes in low-dual hospitals under CJR. We obtained changes in outcomes in high-dual hospitals under CJR by adding β_1 and β_2. We clustered standard errors on MSAs to account for correlation in error terms within MSAs. Our analysis also included sample weights in regressions to correct for any bias caused by stratified sampling (eAppendix 1).

© 2019 Kim H et al. *JAMA Network Open*

eAppendix 5. Comparison of Adjusted Outcomes Between Treatment and Control MSAs Across High- and Low-Dual Hospitals Each Year in the Pre-CJR Period

We compared adjusted outcomes between treatment and control MSAs across high- and low-dual hospitals each year prior to the CJR implementation. If we found no differences in outcomes between treatment and control MSAs *each year* in the pre-CJR period, that would suggest that random selection of MSAs for CJR participation worked well to select comparable treatment and control MSAs. This would also mean that we meet a parallel pre-trend assumption.

Our analysis included joint replacements that occurred prior to the public announcement of CJR model (between 2012 and 2014) in treatment and control MSAs. The unit of regression was each care episode that includes the index hospitalization and 90-day post-discharge care. We stratified the sample to two groups (joint replacements that occurred in high- and low-dual hospitals) and ran a linear ordinary least-squares regression within each of stratified group. We regressed outcomes on an indicator of treatment MSA (vs. control), indicators of year (with year 2012 as a reference group), and interactions between an indicator of treatment MSA and indicators of year. We also adjusted for patient characteristics (type of surgery, presence of major complications or comorbidities during hospital stay, age, and female), hospital characteristics (volume of hip/knee replacements, ownership, major teaching hospital) and MSA characteristics (post-acute care supply per 100,000 people, Medicare Advantage penetration rates, Herfindahl-Hirshman index of market concentration for hip/knee joint replacements in the MSA, indicator of 8 MSA groups based on historical spending and population size) and clustered standard errors on MSAs. We applied Bonferroni correction to outcomes to adjust for multiple testing and to keep familywise error rate at 0.05.

We found no significant differences in adjusted-outcomes when comparing treatment and control MSAs within high-dual hospitals and low-dual hospitals *each year* in the pre-CJR period (see eTable 2a and 2b). Our findings ensure comparability between treatment and control MSAs, and therefore we met a parallel pre-trend assumption.

eAppendix 6. Required Changes in Episode Spending Each Hospital Had/Has to Receive a Bonus (or Avoid a Penalty) in CJR Years 1-5

We assessed required savings that each hospital would have to make in order to receive a CJR bonus (or avoid a penalty) in CJR years 1-5. We calculated them as the difference between the hospital's CJR spending benchmark and historical episode spending.

CJR spending benchmark

CMS sets CJR spending benchmark for each hospital as a weighted average of hospital historical spending and regional historical spending as seen in the following equation:

$$Benchmark_{hosp} = d * \left(w \times HistoricSpending_{hosp} + (1-w) \times HistoricSpending_{region}\right) (1)$$

where $Benchmark_{hosp}$ is the hospital's CJR spending benchmark. $HistoricSpending_{hosp}$ is the hospital's historical episode spending and $HistoricSpending_{region}$ is the regional historical episode spending. w is a weighting factor that determines the impact of hospital historical spending and regional historical spending on CJR spending benchmark. The values of w were 2/3 in CJR years 1-2, 1/3 in CJR year 3, and 0 in CJR years 4-5. d is a deflationary factor and was set to $d = (1 - 0.03)$.

CMS adjusted spending benchmark for hospital wage index, changes in pricing for services, and hospital performance on a composite quality score. We removed the effect of these adjustments from our calculations to focus on the impact of shift toward regional spending in setting CJR spending benchmark. We reported all dollar amounts in 2016 dollars.

We obtained four CJR spending benchmarks for each hospital because CMS sets separate CJR spending benchmark for four groups: (1) elective surgeries without the presence of major complications or comorbidities during the hospital stay (MCC), (2) elective surgeries with MCC, (3) fracture surgeries without MCC, and (4) fracture surgeries with MCC.

Each hospital's spending benchmarks for CJR years 1- 2 are available online.[6]

Hospital historical spending

We obtained hospital historical spending for CJR years 1- 2 by plugging spending benchmarks and regional historical spending into Equation (1). Regional historical spending for CJR years 1-2 is also available online.[6]

We then estimated CJR spending benchmark for years 3-5 by plugging hospital and regional historical spending and weights (that change over time) into Equation (1).

Differences between CJR spending benchmark and hospital historical spending

We calculated the difference between each hospital's historical spending in CJR year 1 with the estimated spending benchmarks for each year. The difference estimated per-episode savings that each hospital would need to achieve to receive a bonus (or avoid a penalty) in each year. Figure 3 in the main manuscript illustrates the mean required savings for high- and low-dual hospitals in CJR years 1-5. We calculated separate required savings for each of four groups (elective vs fracture surgery; with vs without MCC).

© 2019 Kim H et al. *JAMA Network Open*

eTable 1. Definition and Data Source for Outcomes and Explanatory Variables

	Definition	Data source
**Primary outcomes**		
Total spending	Standardized, inflation-adjusted Medicare payments that occurred during episode of care (i.e. index LEJR inpatient hospitalization and 90 day post-discharge period) except payments to durable equipment and hospice care	Medicare 100% claims
Discharge to institutional post-acute care	A binary variable that indicates whether a patient was discharged to institutional post-acute settings (inpatient rehabilitation facility, skilled nursing facility, swing bed, and long-term care hospital)	Medicare 100% claims
Relevant readmission	A binary variable that indicates whether a patient was readmitted to a hospital within 90 day post-discharge period. We excluded irrelevant readmissions based on CMS definition.	Medicare 100% claims
**Secondary outcomes**		
**Standardized, inflation-adjusted spending**		
Index hospitalization	Medicare payments for index inpatient hospitalization.	Medicare 100% claims
Relevant readmission	Medicare payments for relevant readmissions, as defined by CMS, within 90 day post-discharge period.	Medicare 100% claims
Institutional post-acute care	Medicare payments for total institutional post-acute care (skilled nursing facility, inpatient rehab, long-term care hospital, and swing bed) within 90 day post-discharge period	Medicare 100% claims
Skilled nursing facility	Medicare payments for skilled nursing facility use within 90 day post-discharge period.	Medicare 100% claims
Inpatient rehabilitation facility	Medicare payments for inpatient rehabilitation use within 90 day post-discharge period.	Medicare 100% claims
Long-term care hospital	Medicare payments for long-term care use within 90 day post-discharge period.	Medicare 100% claims
Swing bed	Medicare payments for swing bed care use within 90 day post-discharge period.	Medicare 100% claims
Home health agency	Medicare payments for home health care use within 90 day post-discharge period	Medicare 100% claims
Outpatient facility	Medicare payments for outpatient care use within 90 day post-discharge period.	Medicare 100% claims

© 2019 Kim H et al. *JAMA Network Open*

Professional services	Medicare payments for non-institutional care (e.g. non-institutional physician services) within 90 day post-discharge period.	Medicare 100% claims
**Health service use**		
Discharge to home health care	A binary variable that indicates whether a patient was discharged to home health care.	Medicare 100% claims
Discharge to home	A binary variable that indicates whether a patient was discharged to home without any paid care.	Medicare 100% claims
Mean length of stay, Institutional post-acute care facility (days)	Numbers of institutional post-acute care stay days within 90 day post-discharge period.	Medicare 100% claims
Mean length of stay, Index hospitalization (days)	Numbers of index hospitalization stay days	Medicare 100% claims
**Quality of care**		
Complication rates	A binary variable that indicates whether a patient had any complications within 90 day post-discharge period. Taking CMS Hospital Compare's approach, we constructed a composite measure of complications that include the occurrence of heart attack, pneumonia, sepsis, surgical site bleeding, pulmonary embolism, mechanical complications, or periprosthetic joint/wound infection following joint replacement.	Medicare 100% claims
Emergency department visit rates	A binary variable that indicates whether a patient visited an emergency department within 90 day post-discharge period.	Medicare 100% claims
Mortality rates	A binary variable that indicates whether a patient died during care episode (that includes inpatient stay and 90 day post-discharge period).	Medicare 100% claims
Skilled nursing facility star rating 4-5	A binary variable that indicates whether a patient was discharged to skilled nursing facilities with 4-5 stars. We obtained star rating of each facility from publicly available Nursing Home Compare data. The rating was a composite measure of inspection, quality measures, and staffing of skilled nursing facilities, and ranged from 1 to 5, with higher ratings indicating better quality of care. We limited the denominator to patients who were discharged to skilled nursing facilities.	Medicare 100% claims and Nursing Home Compare

© 2019 Kim H et al. *JAMA Network Open*

Timely physical therapy	A binary variable that indicates whether a patient received physical therapy care within 0-2 day after hospital discharge. We limited the denominator to patients who underwent knee replacement surgery and were discharged to home without home health care.	Medicare 100% claims
**Explanatory variables**		
**Key explanatory variables**		
High-dual hospital	A binary variable that indicates whether a hospital was in the top quartile in percentages of dual-eligibles prior to the implementation of CJR.	Medicare 100% claims
Treatment MSAs	A binary variable that indicates whether each hip/knee joint replacement was initiated in one of the MSAs participating in CJR.	Medicare 100% claims and list of hospitals participating in CJR
Post-CJR period	A binary variable that indicates whether each hip/knee joint replacement occurred after the implementation of CJR	Medicare 100% claims
**Other explanatory variables**		
Surgery-level		
Types of surgery	Binary variables that indicate whether a surgery was hip fracture, elective knee, or elective hip replacement surgery based on CMS definition and ICD-9/-10 codes	Medicare 100% claims
Presence of major complications or comorbidities during hospital stay	A binary variable that indicates whether a patient had any major complications or comorbidities during the hospital stay (MS 469 vs MS 470)	Medicare 100% claims
Patient-level		
Age	Binary variables that indicate whether a patient's age was 66-70, 71-75, 76-80, and 81 or above.	Medicare 100% claims
Female	A binary variable that indicates whether a patient was female.	Medicare 100% claims
Hospital fixed effects	Binary variables that indicate each hospital where hip/knee joint replacements occurred	Medicare 100% claims
Year fixed effects	Binary variables that indicate each year when hip/knee joint replacements occurred	Medicare 100% claims
Quarter indicators	Binary variables that indicate quarter when hip/knee joint replacements occurred	Medicare 100% claims

© 2019 Kim H et al. *JAMA Network Open*

eTable 2. Adjusted Outcomes Between Treatment and Control MSAs Among Low-Dual Hospitals and High-Dual Hospitals Each Year in the Pre-CJR Period (2012-2014)

A. Low-Dual Hospitals

	Difference in each outcome between treatment and control MSAs among low-dual hospitals (95% CI) (874 hospitals and 452,891 joint replacements)		
	2012	2013	2014
**Primary Outcomes**			
Total spending, $	-167 (-800 to 466)	76 (-473 to 624)	150 (-518 to 817)
Discharge to institutional post-acute care, %	-0.03 (-0.08 to 0.02)	-0.02 (-0.07 to 0.03)	-0.03 (-0.08 to 0.02)
Relevant readmission rates, %	-0.004 (-0.04 to 0.03)	0.001 (-0.03 to 0.04)	-0.001 (-0.04 to 0.03)
Spending, $			
Index hospitalization	7 (-84 to 97)	22 (-73 to 117)	25 (-74 to 124)
Relevant readmission	-52 (-198 to 95)	36 (-102 to 174)	68 (-88 to 224)
Institutional post-acute care	-145 (-637 to 347)	-69 (-544 to 405)	-16 (-541 to 509)
long-term care hospital	-34 (-144 to 77)	-22 (-125 to 81)	-5 (-104 to 94)
Inpatient rehab facility	-92 (-682 to 498)	-35 (-596 to 525)	-58 (-630 to 515)
Skilled nursing facility	14 (-687 to 715)	28 (-625 to 681)	79 (-552 to 710)
Home health agency	83 (-282 to 447)	124 (-240 to 487)	118 (-261 to 497)
Swing bed	-34 (-274 to 206)	-40 (-297 to 217)	-32 (-298 to 234)
Outpatient facility	-11 (-91 to 68)	0 (-80 to 80)	-15 (-102 to 71)
Professional service	-48 (-258 to 161)	-36 (-230 to 159)	-30 (-231 to 171)
Health Service Use			
Discharge to home health, %	0.04 (-0.03 to 0.11)	0.04 (-0.03 to 0.12)	0.05 (-0.03 to 0.14)
Discharge to home, %	-0.01 (-0.08 to 0.05)	-0.02 (-0.09 to 0.04)	-0.02 (-0.1 to 0.05)
Mean length of stay, Institutional post-acute care facility, days	-0.13 (-1.21 to 0.94)	0.01 (-0.99 to 1.01)	0.15 (-0.87 to 1.16)
Mean length of stay, Index hospitalization, days	0.03 (-0.1 to 0.15)	0.03 (-0.09 to 0.14)	0.03 (-0.11 to 0.17)
Quality of Care, %			
Complication rates	0.0004 (-0.004 to 0.005)	0.0008 (-0.0029 to 0.005)	-0.0014 (-0.0057 to 0.003)
Emergency department visit rates	0.004 (-0.007 to 0.01)	0.004 (-0.008 to 0.02)	0.003 (-0.009 to 0.01)
Mortality rates	-0.0001 (-0.003 to 0.003)	-0.0002 (-0.004 to 0.003)	-0.0005 (-0.003 to 0.002)
Skilled nursing facility star rating 4-5	-0.04 (-0.14 to 0.05)	-0.02 (-0.1 to 0.07)	-0.05 (-0.13 to 0.04)
Timely physical therapy	-0.04 (-0.14 to 0.05)	-0.05 (-0.14 to 0.05)	-0.03 (-0.12 to 0.06)

© 2019 Kim H et al. *JAMA Network Open*

B. High-Dual Hospitals

	Difference in each outcome between treatment and control MSAs among high-dual hospitals (95% CI) (291 hospitals and 69,389 joint replacements)		
	2012	2013	2014
**<u>Primary Outcomes</u>**			
Total spending, $	344 (-948 to 1635)	915 (-296 to 2126)	655 (-522 to 1831)
Discharge to institutional post-acute care, %	0 (-0.09 to 0.08)	0.02 (-0.06 to 0.11)	0.04 (-0.05 to 0.13)
Relevant readmission rates, %	0.01 (-0.06 to 0.08)	0.022 (-0.04 to 0.09)	0.018 (-0.06 to 0.09)
Spending, $			
Index hospitalization	28 (-146 to 202)	67 (-175 to 310)	60 (-109 to 229)
Relevant readmission	212 (-185 to 608)	261 (-82 to 604)	133 (-268 to 534)
Institutional post-acute care	-118 (-1028 to 793)	432 (-376 to 1240)	149 (-815 to 1113)
long-term care hospital	60 (-220 to 340)	83 (-136 to 303)	120 (-90 to 330)
Inpatient rehab facility	39 (-1113 to 1190)	327 (-850 to 1503)	231 (-1114 to 1576)
Skilled nursing facility	-283 (-1743 to 1177)	13 (-1301 to 1327)	-96 (-1461 to 1268)
Home health agency	148 (-302 to 599)	150 (-281 to 581)	181 (-244 to 606)
Swing bed	67 (-346 to 481)	9 (-458 to 476)	-106 (-596 to 384)
Outpatient facility	17 (-116 to 149)	59 (-62 to 180)	24 (-103 to 152)
Professional service	56 (-245 to 358)	-54 (-540 to 432)	108 (-123 to 339)
Health Service Use			
Discharge to home health, %	0.03 (-0.06 to 0.12)	0.02 (-0.07 to 0.1)	0 (-0.1 to 0.1)
Discharge to home, %	-0.03 (-0.09 to 0.02)	-0.04 (-0.1 to 0.01)	-0.04 (-0.11 to 0.02)
Mean length of stay, Institutional post-acute care facility, days	0.01 (-2.14 to 2.16)	0.88 (-1.02 to 2.78)	0.54 (-1.41 to 2.49)
Mean length of stay, Index hospitalization, days	0.23 (0.01 to 0.44)	0.21 (0.02 to 0.4)	0.21 (0 to 0.42)
Quality of Care, %			
Complication rates	0.0019 (-0.0078 to 0.012)	0.0033 (-0.0076 to 0.014)	0.0063 (-0.0061 to 0.019)
Emergency department visit rates	0.002 (-0.02 to 0.03)	0.001 (-0.018 to 0.02)	0.004 (-0.019 to 0.03)
Mortality rates	0.0045 (-0.007 to 0.016)	0.0029 (-0.007 to 0.013)	0.0066 (-0.003 to 0.016)
Skilled nursing facility star rating 4-5	-0.09 (-0.25 to 0.07)	-0.01 (-0.16 to 0.14)	-0.04 (-0.17 to 0.09)
Timely physical therapy	-0.02 (-0.13 to 0.1)	-0.04 (-0.15 to 0.06)	-0.03 (-0.12 to 0.06)

© 2019 Kim H et al. *JAMA Network Open*

eTable 3. Adjusted Hospital-Level Characteristics of High-Dual and Low-Dual Hospitals in the Pre-CJR Period (2012-2014)

**Hospital-level Characteristics**	**High-Dual Hospital (n=291)**	**Low-Dual Hospital (n=874)**	**P Value**
Patient mix, mean (SD)			
Medicaid-enrolled patients, %	18.5(0.3)	4.6(0.2)	<0.001
Medically complex patients, %	13.4(0.2)	11.7(0.1)	<0.001
Nonwhite patients, %	21.6(0.4)	7.4(0.2)	<0.001
Female patients, %	67.3(0.2)	65.1(0.1)	<0.001
Age category			
Age 66-70, %	27.1(0.2)	26.4(0.1)	0.014
Age 71-75, %	25.1(0.2)	24.8(0.1)	0.31
Age 76-80, %	20.5(0.2)	20.6(0.1)	0.58
Age 81 or more, %	27.4(0.2)	28.2(0.1)	0.0045
Hospital characteristics, No. (%)			
Volume of Medicare joint replacements			
Low (11-63)	45.7(1.7)	26.6(0.8)	<0.001
Medium (64-149)	36.3(1.7)	34.6(0.9)	0.39
High (150-1462)	17.9(1.5)	38.8(0.9)	<0.001
Major teaching hospital	22.8(1.5)	16.8(0.7)	<0.001
Ownership type			
For-profit	18.3(1.4)	20.2(0.8)	0.24
Nonprofit	65.0(1.7)	67.4(0.9)	0.23
Public	0.7(0.8)	1.4(0.2)	0.20
Others	15.9(1.2)	10.9(0.6)	<0.001
Operating margin %, mean(SD)	-1.6(0.5)	4.6(0.3)	<0.001

Notes: We adjusted for the CJR model's two risk adjustment components, each hospital's percentage of patients with hip fracture and percentage of patients with major complications or comorbidities. All analyses were done at the hospital level.

© 2019 Kim H et al. *JAMA Network Open*

eTable 4. Differential Changes in Outcomes Before and After Implementation of the CJR Model Among Low-Dual Hospitals and High-Dual Hospitals

A. Low-Dual Hospitals

	Treatment MSAs			Control MSAs			
	(1) Average before CJR	(2) Average after CJR	(3) Unadjusted difference	(4) Average before CJR	(5) Average after CJR	(6) Unadjusted difference	(7) (3)-(6)
**Primary Outcomes**							
Total spending, $	27549	23711	-3838	26843	23734	-3110	-728
Discharge to institutional post-acute care, %	0.48	0.32	-0.16	0.47	0.33	-0.13	-0.03
Relevant readmission rates, %	0.18	0.12	-0.06	0.17	0.13	-0.04	-0.02
Spending, $							
Index hospitalization	13369	12422	-946	13299	12382	-917	-29
Relevant readmission	1428	1292	-136	1338	1207	-131	-5
Institutional post-acute care	6471	4062	-2409	6047	4291	-1756	-653
long-term care hospital	96	52	-45	124	67	-57	13
Inpatient rehab facility	1402	769	-633	1341	960	-381	-252
Skilled nursing facility	4732	3046	-1686	4290	3011	-1279	-406
Home health agency	2180	2043	-138	2106	1987	-120	-18
Swing bed	241	195	-46	291	253	-38	-8
Outpatient facility	634	680	46	661	684	24	22
Professional service	3466	3212	-255	3392	3183	-210	-45
Health Service Use							
Discharge to home health, %	0.36	0.43	0.07	0.34	0.38	0.04	0.03
Discharge to home, %	0.15	0.24	0.09	0.19	0.28	0.09	-0.001
Mean length of stay, Institutional post-acute care facility, days	12.0	7.5	-4.5	11.1	7.6	-3.4	-1.03
Mean length of stay, Index hospitalization, days	4.4	3.7	-0.7	4.3	3.6	-0.7	-0.03
Quality of Care, %							
Complication rates	0.04	0.03	-0.01	0.04	0.03	-0.01	-0.0001
Emergency department visit rates	0.19	0.19	0.00	0.19	0.19	0.00	0.00009
Mortality rates	0.03	0.02	-0.01	0.02	0.02	-0.01	-0.0003
Skilled nursing facility star rating 4-5	0.67	0.73	0.07	0.68	0.70	0.02	0.05
Timely physical therapy	0.21	0.26	0.06	0.23	0.28	0.05	0.007

© 2019 Kim H et al. *JAMA Network Open*

B. High-Dual Hospitals

	Treatment MSAs			Control MSAs			
	(1) Average before CJR	(2) Average after CJR	(3) Unadjusted difference	(4) Average before CJR	(5) Average after CJR	(6) Unadjusted difference	(7) (3)-(6)
**Primary Outcomes**							
Total spending, $	31390	27187	-4202	30950	27386	-3563	-639
Discharge to institutional post-acute care, %	0.60	0.44	-0.15	0.58	0.44	-0.14	-0.02
Relevant readmission rates, %	0.26	0.17	-0.09	0.24	0.19	-0.06	-0.03
Spending, $							
Index hospitalization	13694	12668	-1026	13608	12682	-927	-99
Relevant readmission	2017	1764	-253	1957	1823	-134	-119
Institutional post-acute care	9127	6366	-2761	8939	6726	-2213	-548
long-term care hospital	192	82	-110	255	134	-121	11
Inpatient rehab facility	2269	1208	-1060	2237	1520	-717	-344
Skilled nursing facility	6530	4957	-1573	6101	4717	-1384	-189
Home health agency	2330	2441	111	2314	2268	-46	157
Swing bed	136	120	-17	345	355	10	-26
Outpatient facility	576	610	34	631	701	71	-36
Professional service	3646	3339	-308	3501	3186	-315	7
Health Service Use							
Discharge to home health, %	0.31	0.43	0.13	0.30	0.37	0.07	0.06
Discharge to home, %	0.07	0.12	0.05	0.12	0.19	0.07	-0.028
Mean length of stay, Institutional post-acute care facility, days	16.8	12.0	-4.9	16.2	12.1	-4.1	-0.83
Mean length of stay, Index hospitalization, days	5.0	4.3	-0.7	4.8	4.2	-0.6	-0.05
Quality of Care, %							
Complication rates	0.06	0.05	-0.02	0.06	0.05	-0.01	-0.0043
Emergency department visit rates	0.21	0.21	0.00	0.23	0.23	0.00	-0.00825
Mortality rates	0.04	0.03	-0.01	0.04	0.04	-0.01	-0.0025
Skilled nursing facility star rating 4-5	0.66	0.71	0.06	0.62	0.61	-0.01	0.07
Timely physical therapy	0.13	0.18	0.05	0.16	0.19	0.03	0.020

© 2019 Kim H et al. *JAMA Network Open*

eTable 5. Adjusted Changes in Outcomes Associated With CJR Between Hospitals in the Top and Bottom 3 Quartiles in Disproportionate Patient Percentages (DPP) in CJR Years 1-2 (April 2016 - Dec 2017)

	Hospitals in the top quartile		Hospitals in bottom three quartiles		Top quartile vs bottom three quartiles	
	Changes in outcomes under CJR (95% CI)	P Value	Changes in outcomes under CJR (95% CI)	P Value	Differences in outcomes (95% CI)	P Value
**Primary Outcomes**						
Total spending, $	-944 (-1609 to -279)	0.01	-573 (-947 to -199)	0.00	-371 (-1065 to 323)	0.29
Discharge to institutional post-acute care, %	-0.05 (-0.08 to -0.022)	0.00	-0.02 (-0.04 to 0.004)	0.10	-0.03 (-0.07 to -0.003)	0.03
Relevant readmission rates, %	-0.02 (-0.06 to 0.01)	0.20	-0.02 (-0.04 to 0.01)	0.15	-0.01 (-0.03 to 0.02)	0.61
Spending, $						
Index hospitalization	-68 (-181 to 46)	0.24	-36 (-82 to 10)	0.13	-32 (-152 to 89)	0.60
Relevant readmission	-83 (-221 to 55)	0.24	3 (-70 to 75)	0.94	-86 (-253 to 82)	0.31
Institutional post-acute care	-905 (-1492 to -318)	0.00	-513 (-867 to -160)	0.00	-392 (-902 to 118)	0.13
long-term care hospital	41 (-51 to 132)	0.38	11 (-30 to 52)	0.59	30 (-44 to 103)	0.43
Inpatient rehab facility	-281 (-713 to 152)	0.20	-208 (-431 to 16)	0.07	-73 (-450 to 304)	0.70
Skilled nursing facility	-649 (-1008 to -290)	0.00	-290 (-564 to -16)	0.04	-359 (-721 to 3)	0.052
Home health agency	128 (-98 to 355)	0.26	-12 (-191 to 167)	0.89	141 (-54 to 335)	0.16
Swing bed	-16 (-91 to 59)	0.67	-27 (-81 to 28)	0.33	11 (-78 to 99)	0.81
Outpatient facility	-18 (-57 to 21)	0.36	26 (-1 to 53)	0.06	-44 (-91 to 3)	0.07
Professional service	1 (-140 to 143)	0.98	-40 (-115 to 36)	0.30	41 (-102 to 185)	0.57
Health Service Use						
Discharge to home health, %	0.07 (0.003 to 0.13)	0.04	0.02 (-0.031 to 0.07)	0.44	0.05 (-0.004 to 0.1)	0.07
Discharge to home, %	-0.01 (-0.07 to 0.04)	0.67	0 (-0.04 to 0.05)	0.86	-0.02 (-0.07 to 0.04)	0.56
Mean length of stay, Institutional post-acute care facility, days	-1.5 (-2.3 to -0.7)	0.00	-0.8 (-1.4 to -0.2)	0.01	-0.7 (-1.5 to 0)	0.06

© 2019 Kim H et al. *JAMA Network Open*

Mean length of stay, Index hospitalization, days	-0.08 (-0.21 to 0.05)	0.20	-0.02 (-0.09 to 0.05)	0.58	-0.06 (-0.19 to 0.06)	0.30
Quality of Care, %						
Complication rates	-0.002 (-0.01 to 0.003)	0.53	0 (0 to 0.002)	0.79	-0.001 (-0.01 to 0.004)	0.61
Emergency department visit rates	-0.01 (-0.02 to 0.002)	0.14	0 (0 to 0.006)	0.67	-0.01 (-0.02 to 0.002)	0.11
Mortality rates	-0.002 (-0.01 to 0.002)	0.34	0 (0 to 0.002)	0.80	-0.002 (-0.01 to 0.002)	0.31
Skilled nursing facility star rating 4-5	0.05 (-0.02 to 0.12)	0.13	0.05 (0.01 to 0.1)	0.01	0 (-0.07 to 0.07)	0.98
Timely physical therapy	-0.03 (-0.07 to 0.02)	0.28	0.01 (-0.03 to 0.05)	0.62	-0.04 (-0.09 to 0.02)	0.21

© 2019 Kim H et al. *JAMA Network Open*

eTable 6. Adjusted Changes in Outcomes Associated With CJR Between Hospitals in the Top and Bottom 3 Quartiles in Medicaid Day Percentages in CJR Years 1-2 (April 2016 - Dec 2017)

	Hospitals in the top quartile		Hospitals in bottom three quartiles		Top quartile vs bottom three quartiles	
	Changes in outcomes under CJR (95% CI)	P Value	Changes in outcomes under CJR (95% CI)	P Value	Differences in outcomes (95% CI)	P Value
**Primary Outcomes**						
Total spending, $	-641 (-1019 to -262)	0.00	-520 (-1311 to 272)	0.20	-121 (-984 to 742)	0.78
Discharge to institutional post-acute care, %	-0.02 (-0.05 to 0)	0.05	-0.03 (-0.08 to 0.012)	0.15	0.01 (-0.04 to 0.058)	0.67
Relevant readmission rates, %	-0.02 (-0.04 to 0.01)	0.13	-0.01 (-0.05 to 0.03)	0.58	-0.01 (-0.05 to 0.04)	0.78
Spending, $						
Index hospitalization	-52 (-91 to -12)	0.01	30 (-356 to 416)	0.88	-82 (-474 to 310)	0.68
Relevant readmission	-31 (-95 to 34)	0.35	214 (88 to 341)	0.00	-245 (-381 to -108)	0.001
Institutional post-acute care	-569 (-953 to -185)	0.00	-596 (-1102 to -89)	0.02	27 (-592 to 646)	0.93
long-term care hospital	11 (-37 to 58)	0.65	55 (-14 to 124)	0.12	-44 (-118 to 30)	0.24
Inpatient rehab facility	-237 (-476 to 3)	0.05	-150 (-642 to 342)	0.55	-87 (-588 to 414)	0.73
Skilled nursing facility	-329 (-597 to -60)	0.02	-369 (-794 to 57)	0.09	40 (-412 to 492)	0.86
Home health agency	29 (-148 to 206)	0.75	-176 (-568 to 216)	0.38	205 (-194 to 603)	0.31
Swing bed	-14 (-61 to 33)	0.56	-132 (-307 to 43)	0.14	118 (-56 to 292)	0.18
Outpatient facility	19 (-6 to 43)	0.14	16 (-52 to 83)	0.65	3 (-67 to 73)	0.93
Professional service	-37 (-113 to 38)	0.33	-8 (-138 to 121)	0.90	-29 (-170 to 112)	0.69
Health Service Use						
Discharge to home health, %	0.03 (-0.019 to 0.08)	0.22	-0.01 (-0.139 to 0.12)	0.89	0.04 (-0.088 to 0.17)	0.54
Discharge to home, %	0 (-0.04 to 0.03)	0.81	0.06 (-0.06 to 0.17)	0.35	-0.06 (-0.17 to 0.05)	0.29
Mean length of stay, Institutional post-acute care facility, days	-0.9 (-1.5 to -0.3)	0.00	-1 (-1.7 to -0.3)	0.00	0.2 (-0.7 to 1.1)	0.68

© 2019 Kim H et al. *JAMA Network Open*

Mean length of stay, Index hospitalization, days	-0.03 (-0.1 to 0.04)	0.41	-0.05 (-0.21 to 0.1)	0.49	0.02 (-0.14 to 0.19)	0.76
Quality of Care, %						
Complication rates	-0.001 (0 to 0.002)	0.56	0 (0 to 0.003)	0.90	0 (0 to 0.004)	0.84
Emergency department visit rates	0 (-0.01 to 0.004)	0.68	0.01 (-0.01 to 0.024)	0.42	-0.01 (-0.03 to 0.01)	0.38
Mortality rates	0 (0 to 0.002)	0.86	0 (0 to 0.002)	0.73	0 (0 to 0.003)	0.88
Skilled nursing facility star rating 4-5	0.05 (0.01 to 0.09)	0.02	0.17 (0.02 to 0.33)	0.03	-0.13 (-0.28 to 0.03)	0.11
Timely physical therapy	0 (-0.04 to 0.04)	0.86	0 (-0.05 to 0.06)	0.91	0 (-0.06 to 0.06)	0.99

© 2019 Kim H et al. *JAMA Network Open*

eTable 7. Adjusted Changes in Outcomes Associated With CJR Between Hospitals in the Top and Bottom 3 Quartiles in Percentage of Patients in an Area of High Poverty in CJR Years 1-2 (April 2016 - Dec 2017)

	Hospitals in the top quartile		Hospitals in bottom three quartiles		Top quartile vs bottom three quartiles	
	Changes in outcomes under CJR (95% CI)	P Value	Changes in outcomes under CJR (95% CI)	P Value	Differences in outcomes (95% CI)	P Value
**Primary Outcomes**						
Total spending, $	-1211 (-1794 to -628)	0.00	-458 (-830 to -86)	0.02	-753 (-1344 to -161)	0.013
Discharge to institutional post-acute care, %	-0.05 (-0.08 to -0.017)	0.00	-0.02 (-0.04 to 0.008)	0.20	-0.03 (-0.07 to 0.002)	0.06
Relevant readmission rates, %	-0.03 (-0.06 to 0)	0.05	-0.01 (-0.03 to 0.01)	0.21	-0.02 (-0.04 to 0.01)	0.13
Spending, $						
Index hospitalization	-103 (-202 to -3)	0.04	-29 (-80 to 23)	0.27	-74 (-182 to 34)	0.18
Relevant readmission	-61 (-202 to 80)	0.39	9 (-61 to 79)	0.80	-70 (-233 to 93)	0.40
Institutional post-acute care	-1141 (-1681 to -601)	0.00	-411 (-762 to -60)	0.02	-730 (-1182 to -279)	0.002
long-term care hospital	45 (-76 to 166)	0.46	3 (-26 to 32)	0.85	42 (-69 to 154)	0.46
Inpatient rehab facility	-468 (-829 to -106)	0.01	-175 (-414 to 64)	0.15	-292 (-656 to 71)	0.11
Skilled nursing facility	-637 (-1076 to -198)	0.00	-234 (-481 to 13)	0.06	-403 (-776 to -29)	0.03
Home health agency	154 (-121 to 429)	0.27	-28 (-198 to 142)	0.75	182 (-48 to 412)	0.12
Swing bed	-82 (-187 to 23)	0.13	-4 (-56 to 47)	0.87	-77 (-193 to 39)	0.19
Outpatient facility	4 (-40 to 48)	0.87	24 (-3 to 50)	0.08	-20 (-69 to 29)	0.42
Professional service	-64 (-162 to 34)	0.20	-23 (-101 to 54)	0.55	-41 (-138 to 57)	0.41
Health Service Use						
Discharge to home health, %	0.07 (-0.009 to 0.15)	0.08	0.02 (-0.034 to 0.06)	0.54	0.05 (-0.013 to 0.12)	0.12
Discharge to home, %	-0.01 (-0.08 to 0.06)	0.77	0 (-0.04 to 0.04)	0.87	-0.01 (-0.08 to 0.05)	0.69
Mean length of stay, Institutional post-acute care facility, days	-1.8 (-2.7 to -0.9)	0.00	-0.6 (-1.1 to -0.1)	0.03	-1.2 (-2 to -0.4)	0.003

© 2019 Kim H et al. *JAMA Network Open*

Mean length of stay, Index hospitalization, days	-0.07 (-0.2 to 0.07)	0.33	-0.02 (-0.09 to 0.05)	0.53	-0.04 (-0.19 to 0.1)	0.54
Quality of Care, %						
Complication rates	-0.002 (-0.01 to 0.002)	0.38	0 (0 to 0.002)	0.80	-0.002 (-0.01 to 0.003)	0.49
Emergency department visit rates	0 (-0.01 to 0.008)	0.73	0 (0 to 0.005)	0.92	0 (-0.01 to 0.008)	0.69
Mortality rates	-0.001 (0 to 0.002)	0.46	0 (0 to 0.002)	0.84	-0.001 (0 to 0.002)	0.40
Skilled nursing facility star rating 4-5	0.07 (0 to 0.14)	0.07	0.05 (0.01 to 0.09)	0.03	0.02 (-0.06 to 0.1)	0.61
Timely physical therapy	-0.04 (-0.1 to 0.01)	0.14	0.02 (-0.02 to 0.06)	0.41	-0.06 (-0.13 to 0.01)	0.08

© 2019 Kim H et al. *JAMA Network Open*

eTable 8. Intention-to-Treat Approach: Adjusted Changes in Spending, Health Service Use, and Quality of Care Associated With the CJR Model Between High-Dual and Low-Dual Hospitals in CJR Years 1-2 (April 2016 - Dec 2017)

	High-dual hospitals		Low-dual hospitals		High- vs low-dual hospitals	
	Changes in outcomes under CJR (95% CI)	P Value	Changes in outcomes under CJR (95% CI)	P Value	Differences in outcomes (95% CI)	P Value
**Primary Outcomes**						
Total spending, $	-822 (-1488 to -155)	0.02	-478 (-822 to -135)	0.01	-343 (-1003 to 316)	0.31
Discharge to institutional post-acute care, %	-0.03 (-0.06 to 0.002)	0.07	-0.02 (-0.04 to 0.003)	0.09	-0.01 (-0.04 to 0.02)	0.54
Relevant readmission rates, %	-0.028 (-0.08 to 0.02)	0.28	-0.015 (-0.03 to 0.001)	0.07	-0.013 (-0.06 to 0.03)	0.55
Spending, $						
Index hospitalization	-81 (-203 to 41)	0.19	-32 (-78 to 15)	0.18	-49 (-178 to 80)	0.45
Relevant readmission	-156 (-342 to 30)	0.10	22 (-36 to 81)	0.45	-178 (-373 to 16)	0.07
Institutional post-acute care	-734 (-1378 to -89)	0.03	-468 (-783 to -153)	0.00	-266 (-802 to 271)	0.33
long-term care hospital	42 (-50 to 134)	0.37	16 (-24 to 55)	0.43	26 (-57 to 110)	0.53
Inpatient rehab facility	-267 (-695 to 160)	0.22	-232 (-438 to -25)	0.03	-36 (-421 to 350)	0.86
Skilled nursing facility	-440 (-899 to 20)	0.06	-251 (-494 to -9)	0.04	-189 (-599 to 222)	0.37
Home health agency	173 (-76 to 422)	0.17	6 (-149 to 161)	0.94	167 (-25 to 360)	0.09
Swing bed	-69 (-158 to 21)	0.13	-1 (-56 to 55)	0.98	-68 (-173 to 37)	0.20
Outpatient facility	-24 (-68 to 21)	0.29	23 (-1 to 47)	0.07	-47 (-94 to 0.3)	0.05
Professional service	0 (-156 to 155)	1.00	-30 (-98 to 38)	0.38	30 (-126 to 185)	0.71
Health Service Use						
Discharge to home health, %	0.06 (0 to 0.11)	0.04	0.02 (-0.02 to 0.07)	0.31	0.04 (-0.01 to 0.08)	0.14
Discharge to home, %	-0.01 (-0.05 to 0.02)	0.40	0 (-0.04 to 0.04)	0.90	-0.01 (-0.05 to 0.03)	0.56
Mean length of stay, Institutional post-acute care facility, days	-1.2 (-2.28 to -0.14)	0.03	-0.68 (-1.19 to -0.17)	0.01	-0.53 (-1.44 to 0.39)	0.26
Mean length of stay,	-0.03 (-0.14 to 0.09)	0.63	-0.02 (-0.09 to 0.04)	0.47	-0.004 (-0.12 to 0.11)	0.94

© 2019 Kim H et al. *JAMA Network Open*

Index hospitalization, days						
Quality of Care, %						
Complication rates	-0.0021 (-0.007 to 0.003)	0.41	-0.0003 (-0.0026 to 0.002)	0.81	-0.0018 (-0.0073 to 0.004)	0.52
Emergency department visit rates	-0.008 (-0.021 to 0.005)	0.21	0.001 (-0.004 to 0.005)	0.77	-0.009 (-0.022 to 0.004)	0.17
Mortality rates	-0.0018 (-0.006 to 0.003)	0.44	-0.0003 (-0.002 to 0.001)	0.71	-0.0015 (-0.006 to 0.003)	0.52
Skilled nursing facility star rating 4-5	0.08 (0.01 to 0.15)	0.02	0.05 (0.01 to 0.1)	0.02	0.03 (-0.05 to 0.1)	0.47
Timely physical therapy	0.03 (-0.05 to 0.11)	0.44	0 (-0.04 to 0.04)	0.98	0.03 (-0.06 to 0.12)	0.47

© 2019 Kim H et al. *JAMA Network Open*

eTable 9. Required Change in Episode Spending Each Hospital Had/Has to Receive a Bonus (or Avoid a Penalty) in CJR Years 1-5

	Elective without MCC		Elective with MCC		Hip fracture without MCC		Hip fracture with MCC	
Year	High-dual	Low-dual	High-dual	Low-dual	High-dual	Low-dual	High-dual	Low-dual
1	-887	-89	-1,245	-140	-1,608	-206	-2,231	-215
2	-887	-89	-1,245	-140	-1,608	-206	-2,231	-215
3	-1,775	-178	-2,491	-280	-3,217	-412	-4,463	-429
4	-2,662	-267	-3,736	-420	-4,825	-618	-6,694	-644
5	-2,662	-267	-3,736	-420	-4,825	-618	-6,694	-644

Notes: MCC is the occurrence of major complications or comorbidities during the hospital stay.

© 2019 Kim H et al. *JAMA Network Open*
